# Monoclonal antibodies against lipopolysaccharide protect against *Pseudomonas aeruginosa* challenge in mice

**DOI:** 10.3389/fcimb.2023.1191806

**Published:** 2023-06-22

**Authors:** Jason Kang, Margalida Mateu-Borrás, Hunter L. Monroe, Emel Sen-Kilic, Sarah Jo Miller, Spencer R. Dublin, Annalisa B. Huckaby, Evita Yang, Gage M. Pyles, Mason A. Nunley, Josh A. Chapman, Md Shahrier Amin, F. Heath Damron, Mariette Barbier

**Affiliations:** ^1^ Department of Microbiology, Immunology, and Cell Biology, West Virginia University, Morgantown, WV, United States; ^2^ Vaccine Development Center, West Virginia University Health Sciences Center, Morgantown, WV, United States; ^3^ Department of Pathology, Anatomy, and Laboratory Medicine, West Virginia University, Morgantown, WV, United States

**Keywords:** *Pseudomonas aeruginosa*, monoclonal antibody (mAb), anti-lipopolysaccharide antibody, O5, ammonium metavanadate, sepsis, pneumonia, immunotherapeutics

## Abstract

*Pseudomonas aeruginosa* is a common cause of hospital-acquired infections, including central line-associated bloodstream infections and ventilator-associated pneumonia. Unfortunately, effective control of these infections can be difficult, in part due to the prevalence of multi-drug resistant strains of *P. aeruginosa*. There remains a need for novel therapeutic interventions against *P. aeruginosa*, and the use of monoclonal antibodies (mAb) is a promising alternative strategy to current standard of care treatments such as antibiotics. To develop mAbs against *P. aeruginosa*, we utilized ammonium metavanadate, which induces cell envelope stress responses and upregulates polysaccharide expression. Mice were immunized with *P. aeruginosa* grown with ammonium metavanadate and we developed two IgG2b mAbs, WVDC-0357 and WVDC-0496, directed against the O-antigen lipopolysaccharide of *P. aeruginosa*. Functional assays revealed that WVDC-0357 and WVDC-0496 directly reduced the viability of *P. aeruginosa* and mediated bacterial agglutination. In a lethal sepsis model of infection, prophylactic treatment of mice with WVDC-0357 and WVDC-0496 at doses as low as 15 mg/kg conferred 100% survival against challenge. In both sepsis and acute pneumonia models of infection, treatment with WVDC-0357 and WVDC-0496 significantly reduced bacterial burden and inflammatory cytokine production post-challenge. Furthermore, histopathological examination of the lungs revealed that WVDC-0357 and WVDC-0496 reduced inflammatory cell infiltration. Overall, our results indicate that mAbs directed against lipopolysaccharide are a promising therapy for the treatment and prevention of *P. aeruginosa* infections.

## 1 Introduction

*Pseudomonas aeruginosa* is a ubiquitous, Gram-negative pathogen that thrives in many natural environments and habitats (Diaz et al., 2018; Laborda et al., 2021). As part of the pseudomonad genus, *P. aeruginosa* has minimal nutritional requirements and can persist on frequently contacted surfaces found in hospital rooms and medical equipment (Pachori et al., 2019). Consequently, *P. aeruginosa* is one of the most common sources for nosocomial infections and can cause serious illness in immunocompromised patients, such as ventilator-associated pneumonia (VAP) and primary bloodstream infections (Spagnolo et al., 2021). Among Gram-negative bacteria, *P. aeruginosa* is the most common cause of VAP (Sader et al., 2014), and second most common cause of primary bloodstream infections (Richards et al., 1999). The healthcare and socioeconomic burden from VAP and primary bloodstream infections are significant, and these complications are associated with increased mortality, greater hospital costs, and longer lengths of stay (Chastre and Fagon, 2002; Brun-Buisson et al., 2003; Warren et al., 2003; Wisplinghoff et al., 2004; Chiang et al., 2021).

Although the selection of appropriate antibiotics is central in improving patient outcomes, the development of antibiotic resistance by *P. aeruginosa* has presented an enormous challenge for healthcare providers. *P. aeruginosa* can develop resistance to multiple classes of antibiotics through intrinsic and acquired mechanisms of resistance, which include multi-drug efflux pumps, antibiotic-modifying enzymes, and the horizontal transfer of mobile genetic elements (Pang et al., 2019). Of concern is the prevalence of multidrug-resistant (MDR) *P. aeruginosa* (resistance to three or more drug classes). In a review by Puzniak et al., MDR *P. aeruginosa* was accountable for 72.3% of all *P. aeruginosa* infections (Puzniak et al., 2021). In 2017, the CDC reported that infections from MDR *P. aeruginosa* were responsible for 32,600 hospitalizations, 2,700 deaths, and $767 million in attributable healthcare costs (Centers for Disease Control, 2019). As a result, MDR *P. aeruginosa* has been listed as a serious public health threat that requires immediate action (Centers for Disease Control, 2019). Although there is a need for new therapeutic agents against MDR *P. aeruginosa*, there are few drugs in the developmental pipeline (Butler et al., 2022).

An important virulence factor in the pathogenesis of *P. aeruginosa* infections is lipopolysaccharide (LPS), which promotes evasion of host defenses and establishment of infection (Huszczynski et al., 2020). LPS is also a potent stimulator of the innate immune system and can generate strong inflammatory responses. This host response is mediated by the activation of toll-like receptor 4 (TLR4), which triggers production of pro-inflammatory cytokines, such as IL-1β, IL-6 and TNF-α (Kawai et al., 2001). However, if left unregulated, an excessive production of pro-inflammatory cytokines can eventually lead to severe inflammatory conditions, such as sepsis and multi-organ failure (Ulloa and Tracey, 2005; Kell and Pretorius, 2015). The passive administration of monoclonal antibodies (mAbs) can provide rapid protection against *P. aeruginosa* infections, and is ideal for susceptible individuals who are immunocompromised or have acute exposure to the pathogen (Krause et al., 1997). Numerous pre-clinical studies have demonstrated that the administration of anti-LPS mAbs against the O-antigen of *P. aeruginosa* reduces bacterial burden and improves survival in multiple animal models of infection (Akiyama et al., 2000; Hemachandra et al., 2001; Horn et al., 2010; Xie et al., 2010). In a phase IIa clinical trial with Panobacumab, an anti-LPS mAb against serotype O11, it was shown that adjunctive therapy with standard of care antibiotics improved clinical resolution of patients with confirmed O11 pneumonia (Que et al., 2014). Though there are currently no FDA approved mAbs for *P. aeruginosa* infections, the promising outcomes of these pre-clinical and clinical studies support the further development of anti-LPS mAbs against *P. aeruginosa*.

One method for upregulating bacterial cell surface polysaccharides is to use ammonium metavanadate (AMV), which is a phosphatase inhibitor that induces cell envelope stress responses (Damron et al., 2011). When *P. aeruginosa* is cultured on Pseudomonas isolation agar (PIA) supplemented with AMV (PIAAMV), numerous genotypic changes occur, and the resultant phenotype closely resembles other clinical isolates that have undergone similar envelope stress responses. Notably, we had previously demonstrated that when *P. aeruginosa* PAO1 was cultured on PIAAMV, a large number of genes associated with LPS increased expression levels, including the entire O-antigen operon (*wzz* to *wbpM*) (Damron et al., 2013). In addition, the lipid A moiety was altered to include a palmitate group (Damron et al., 2011), which is a common modification among cystic fibrosis isolates (Ernst et al., 2007). This hexa-acylated form of lipid A has been shown to increase recognition of TLR4 by *P. aeruginosa* (Hajjar et al., 2002). Interestingly, in a survival study of mice challenged with *P. aeruginosa* PAO1 cultured on PIA or PIAAMV, there was an improved survival of mice in the PIAAMV group (Damron et al., 2013). Based on these findings, we hypothesized that the predominant antibody response in mice challenged with *P. aeruginosa* cultured on PIAAMV was likely to outer membrane polysaccharides and the development of these antibodies would lead to a protective therapy against *P. aeruginosa* infections.

Herein, we describe the discovery of mAbs that are highly protective against *P. aeruginosa* infections. WVDC-0357 and WVDC-0496 were derived from mice challenged with *P. aeruginosa* PAO1 cultured on PIAAMV. We demonstrate that these mAbs are highly protective in clinically relevant models of sepsis and acute pneumonia. Our data demonstrate that WVDC-0357 and WVDC-0496 reduce the number of viable bacteria and protect against *P. aeruginosa* challenge in multiple models of infection. Furthermore, protection provided by WVDC-0357 and WVDC-0496 was linked with a decreased inflammatory cytokine profile. Altogether, these results support the translation and use of WVDC-0357 and WVDC-0496 against *P. aeruginosa* infections.

## 2 Materials and methods

### 2.1 Generation of hybridomas

Six-week-old CD-1 mice (Charles River, strain 022) were primed and boosted with an intranasal (IN) dose of 1x10^7^ CFU of live *P. aeruginosa* strain PAO1 Vasil (Barton et al., 1996) grown on Pseudomonas isolation agar (PIA) supplemented with 0.3 mM ammonium metavanadate (AMV). After 60 d, mice were euthanized and the spleen was harvested for hybridoma generation. Hybridomas were generated by fusing 1x10^7^ splenocytes with 1x10^7^ SP2/O-Ag14 myeloma cells (ATCC CRL-1581™) using the ECM2001+ electrofusion apparatus (BTX). Fused cells were cultured in ClonaCell-HY medium C (STEMCELL Technologies) and incubated overnight at 37°C and 5% CO_2_. The next day, cells were centrifuged and cultured in ClonaCell-HY medium D (STEMCELL Technologies), which is a semi-solid medium containing hypoxanthine-aminopterin-thymidine (HAT). After ten days of incubation, single hybridoma colonies were selected and plated in 96-well plates containing ClonaCell-HY medium E (STEMCELL Technologies). Supernatant from the well plates were screened with ELISA to identify IgG antibodies against *P. aeruginosa*. Select hybridoma colonies were produced at high densities using a hollow fiber bioreactor (FiberCell Systems) containing Dulbecco’s Modified Eagle’s Medium (DMEM) supplemented with serum-free chemically defined medium for high density cell culture (CDM-HD). Antibodies from the bioreactor harvests were purified with the ÄKTA pure chromatography system (Cytiva) using a HiScreen Fibro™ Prism A column (Cytiva) and HiScale™ 16/20 column (Cytiva). Purified mAbs were eluted in 0.2M sodium phosphate buffer (pH 7.4) and antibody concentrations were determined by measuring the Protein A280 with an E1% value of 14.00. The isotype of each antibody was determined using the Pierce Rapid Antibody Isotyping Kit (ThermoFisher Scientific, #26179).

### 2.2 Enzyme-linked immunosorbent assay

ELISA assays were performed using a panel of *P. aeruginosa* strains (**Supplementary Table 1**). *In silico* serogrouping of *P. aeruginosa* strains were performed with whole genome sequences using the *Pseudomonas aeruginosa* serotyper (PAst) (Thrane et al., 2016). *P. aeruginosa* strains were grown on PIA overnight at 37°C. Bacterial plates were swabbed into sterile PBS and adjusted to a bacterial density that corresponded to 1x10^9^ CFU/ml. To prepare ELISA plates, 96-well microtiter plates (ThermoFisher Scientific, #15041) were coated with 50 μL of bacteria containing 5x10^7^ CFU in phosphate buffered saline (PBS) and incubated overnight at 4°C. Wells were washed three times with PBS with 0.05% Tween 20 (PBS-T) and blocked with 2% bovine serum albumin (BSA) (Research Products International, #A30075) overnight at 4°C. Primary antibodies (serum, hybridoma supernatant, WVDC-0357, or WVDC-0496) diluted in 2% BSA were applied to the wells and incubated for 1 h at 37°C. Wells were washed four times with PBS-T. For plates developed with tetramethyl benzidine (TMB), 100 µl of anti-IgG secondary antibody conjugated to horseradish peroxidase (HRP) (Novus biological, #NBP1-75130) at a dilution of 1:2000 in 2% BSA was applied to each well for 1 h at 37°C. After incubation, wells were washed five times with PBS-T and 100 µL of a TMB solution (BioLegend, #421101) was added to each well. After 10-15 min, the reaction was stopped by adding 50 µl of 2N sulfuric acid and absorbance was read at 450 nm using a SpectraMax i3 plate reader (Molecular Devices LLC). For plates developed with Pierce p-Nitrophenyl Phosphate (PNPP), 100 µl of anti-IgG secondary antibody conjugated to alkaline phosphatase (AP) (Southern Biotech, #1030-04) at a dilution of 1:2000 in 2% BSA was applied to each well for 1 h at 37°C. After incubation, wells were washed five times with PBS-T and 100 µL of a PNPP solution (ThermoFisher Scientific, #34045) was added to each well. After 30 min, the absorbance was read at 405 nm using a SpectraMax i3 plate reader. Absorbance was plotted against antibody concentration and the area under the curve (AUC) was calculated using Graphpad Prism.

### 2.3 Immunoblotting

To identify the target of antibody binding, western blot analysis was performed with *P. aeruginosa* strain PAO1 treated with proteinase K or sodium periodate as demonstrated by Nielsen et al. (2017). In addition, western blot analysis was performed with strains from the University of Washington *P. aeruginosa* PAO1 transposon mutant library (Held et al., 2012). Outer membrane fractions were prepared based on methods described by Hernández-Allés et al. (1999). *P. aeruginosa* PAO1 outer membrane fractions were treated with 0.2 mg/ml proteinase K (Invitrogen, #25530049) or with 20 mM sodium periodate (ThermoFisher Scientific, #20504) at 37°C for 2 h. Afterwards, 2 μg of sample was added to Laemmli buffer (Sigma-Aldrich, #S3041) and boiled at 95°C for 5 min. Samples were then loaded into 12% polyacrylamide gels (ThermoFisher, #XP00122BOX) and resolved by gel electrophoresis. Samples were transferred onto nitrocellulose membranes (ThermoFisher, #IB23001) using the iBlot™ 2 Gel Transfer Device (ThermoFisher, #IB21001) and blocked in 5% skim milk overnight. Membranes were treated with 2 µg/ml of WVDC-0357 or WVDC-0496 in 1% skim milk for 1 h at room temperature. Membranes were then washed three times with PBS-T and treated with anti-IgG secondary antibody conjugated to HRP (Novus biological, #NBP1-75130) at a 1:5000 dilution in 1% skim milk for 1 h at room temperature. Membranes were washed three times with PBS-T and developed using Pierce™ ECL Western Blotting Substrate (ThermoFisher, #32106). Chemiluminescent images of the blots were taken using a Chemidoc Touch Imaging System (Bio-Rad, #1708370).

### 2.4 Flow cytometry binding assay

To demonstrate binding of WVDC-0357 and WVDC-0496 to *P. aeruginosa* with flow cytometry, we used methods described previously (DeJong et al., 2022). Briefly, *P. aeruginosa* strain PAO1 was cultured to mid-log phase growth, washed, and concentrated in PBS. Approximately 1x10^7^ CFUs of bacteria were incubated with 10 µg/ml of an IgG2b isotype control mAb (BioXcell, #BE0086), WVDC-0357, or WVDC-0496 for 30 min at 37°C. Samples were washed once with PBS and incubated with 1.25 µg/ml Fc block (BD Biosciences, #553142) for 15 min at 4°C. Afterwards, 2.5 µg/ml of anti-IgG secondary antibody conjugated to APC-Cy7 (BioLegend, #405316) was added to samples and incubated for 30 min at 4°C. Samples were washed twice with PBS and *P. aeruginosa* was stained with BacLight Green bacterial stain (Invitrogen, #B35000). Samples were analyzed on a Guava^®^ easyCyte 12HT flow cytometer (Luminex). Flow cytometry results were analyzed using FlowJo™ v10.8 Software (BD Life Sciences).

### 2.5 Measurement of antibody binding kinetics

To determine the binding kinetics of WVDC-0357 and WVDC-0496, a flow cytometry binding assay was performed. To allow bacteria-antibody complexes to form, 1x10^7^ CFU of *P. aeruginosa* PAO1 was incubated with varying concentrations of an IgG2b isotype control mAb, WVDC-0357, or WVDC-0496 for 1 h at 4°C. Samples were washed once and resuspended in 100 μL of PBS. Afterwards, bacteria were exposed to a 1:400 dilution of rabbit anti-mouse anti-IgG conjugated to Alexa-Fluor 488 (ThermoFisher Scientific, #A11059). Samples were washed once in PBS and fixed in 0.4% v/v paraformaldehyde (Sigma-Aldrich, #P6148) overnight at 4°C. Following fixation, samples were washed twice and resuspended in 300 μL of PBS. Samples were analyzed using a BD Fortessa and results were analyzed using FlowJo™ v10.8 Software. Data were fitted with a non-linear regression fit to determine the dissociation constant (K_D_)

### 2.6 Assessment of bacterial clearance

To assess the capability of WVDC-0357 and WVDC-0496 to facilitate clearance of bacteria, a modified version of an opsonophagocytosis killing assay was performed as described by Paschall et al. (2019). *P. aeruginosa* strain PAO1 was cultured to mid-log phase growth, washed, and resuspended to 1x10^4^ CFU/ml in opsonization buffer B (OBB; PBS with Ca^2+^ and Mg^2+^, supplemented with 5% heat-inactivated fetal bovine serum and 0.1% gelatin). In round bottom 96-well plates (Fisherbrand, #FB012932), 50 µl of bacteria and 50 µl of treatment (PBS, IgG2b isotype control mAb, WVDC-0357, or WVDC-0496 at varying concentrations) were added to each well and incubated for 1 h at room temperature with shaking at 120 rpm. Afterwards, J774A.1 macrophages (ATCC, #TIB-67™), baby rabbit complement (Pel-Freez Biologicals, #31061), J774A.1 macrophages with baby rabbit complement, or OBB was added to each well and incubated for 1 h at 37°C with shaking at 120 rpm. A 1000:1 effector-to-target cell ratio of macrophages to bacterium was used. After incubation, plates were put on ice for 15 min to stop the reaction. Samples were serially diluted and plated on PIA. Plates were incubated overnight at 37°C. Remaining bacterial colonies were enumerated and expressed as percent killings relative to assays run without antibody.

### 2.7 Assessment of agglutination and turbidity in *P. aeruginosa* cultures

In UV-transparent cuvettes (BrandTech, #759015), an overnight culture of *P. aeruginosa* PAO1 was diluted 1:50 in lysogeny broth and treated 100 µg/ml of IgG2b isotype control, WVDC-0357, or WVDC-0496 mAb. Cuvettes were incubated statically at 37°C and the absorbance at 600 nm was read at time points up to 24 h. To visualize turbidity of growth cultures, cuvettes were photographed at 24 h. To visualize and quantify bacterial agglutination, *P. aeruginosa* PAO1 was cultured to mid-log phase growth, washed, and concentrated in PBS. Approximately 5x10^7^ CFUs of bacteria were stained with BacLight Red bacterial stain (Invitrogen, #B35001) for 15 min at room temperature, pelleted, and resuspended in PBS. Antibody was added at a final concentration of 100 µg/ml and incubated statically at 37°C for 2 h. Samples were placed on glass slides, mounted with cover slips, and sealed with nail polish. Fluorescent images were taken using an EVOS FL imaging system (ThermoFisher, #AMEFC4300). For aggregation analysis, 20 randomly imaged areas were analyzed for number and size of aggregates using a custom pipeline in CellProfiler 4.2.4.

### 2.8 Transmission electron microscopy imaging

To label and visualize external structures on the surface of *P. aeruginosa* with antibodies, we followed an immunogold labeling method described by Javed et al. (2015). Briefly, formvar-coated copper (300 mesh) grids (Sigma-Aldrich, #TEM-FCF300CU50) were floated on drops of an overnight culture of *P. aeruginosa* for 1 h. The grids were washed once in PBS and fixed in 2.5% paraformaldehyde for 20 min. The grids were washed three times in PBS and placed in blocking solution (5% BSA in 0.05% PBS-T). Grids were then incubated with 10 µg/ml of an IgG2b isotype control mAb, WVDC-0357, or WVDC-0496 for 45 min. Grids were washed three times in blocking solution and incubated with goat anti-mouse IgG conjugated to 10 nm gold (Sigma-Aldrich, #G7652) diluted 1:50 in blocking solution for 45 min. The grids were then washed three times in blocking solution, PBS, and milli-Q water. The grids were air dried on Whatman filter paper (Cytiva, #1001085). The grids were examined using a transmission electron microscope (JEOL JEM-2100) equipped with a Gatan OneView camera.

### 2.9 Murine challenge models

To assess protection in convalescent mice previously challenged with *P. aeruginosa* PAO1 cultured on PIAAMV, an acute pneumonia model was used. *P. aeruginosa* strain PAO1 was cultured on PIAAMV overnight at 37°C. Bacterial plates were swabbed and resuspended in PBS. Based on the optical density at 600 nm, a dose was prepared at 1.4x10^7^ CFU per 20 μL in PBS. Six-week old, female CD-1 mice were anesthetized with ketamine (77 mg/kg) (Patterson Veterinary, #07-803-6637) and xylazine (7.7 mg/kg) (Patterson Veterinary, #07-909-1939) in 0.9% saline. Anesthetized mice were IN challenged by pipetting 10 μL of bacteria in each nostril. Mice were allowed to convalesce for 34 days. A dose of *P. aeruginosa* PAO1 cultured on PIA was prepared at 1.4x10^8^ CFU per 20 μL. Convalescent mice and age-matched naïve CD-1 mice were anesthetized and IN challenged with the prepared dose. Mice were euthanized at 16 h post-challenge with an intraperitoneal (IP) injection of Euthasol (390 mg/kg) (Patterson Veterinary, #07-805-9296). Blood was collected *via* cardiac puncture and placed in serum separator tubes (BD, #365967). The lung was aseptically removed, homogenized, and plated to determine CFU loads. The nasal cavity was flushed with PBS and plated to determine CFU loads in the nares.

To evaluate the efficacy of WVDC-0357 and WVDC-0496 in a sepsis model of infection, nine-week old female CD-1 mice were IP administered either an IgG2b isotype control mAb, serum from heat-killed *P. aeruginosa* whole-cell vaccinated (*Pa* WCV) mice (Sen-Kilic et al., 2021; Blackwood et al., 2022), WVDC-0357, or WVDC-0496. After 12 h, mice were challenged with an IP 5x10^5^ CFU dose of *P. aeruginosa* PAO1. To determine survival, mice were monitored for up to 5 days after challenge. Health of the mice were scored based on six criteria: appearance, activity, eye closure, respiration quality, body temperature, and body weight loss. For each category, mice were scored from 0-4, where 0 represented no symptoms, and 4 represented the most severe phenotype (Shrum et al., 2014). Mice were humanely euthanized if mice reached a score of 4 in any category or a total score of 12 and above.

To look at correlates of protection in the sepsis model, mice were passively immunized as before, and euthanized at 6 h post-challenge for tissue collection. Mice were euthanized with an IP injection of Euthasol (390 mg/kg). Blood was collected *via* cardiac puncture and placed in serum separator tubes. The kidney and spleen were aseptically removed. The right kidney was dounce homogenized. The spleen was homogenized in a gentleMACS C tube (Miltenyi Biotec, #130-093-237). To quantify bacterial burden, blood, kidney, and spleen samples were serially diluted in PBS, plated on PIA, and incubated overnight at 37 °C for CFU enumeration.

To assess the efficacy of WVDC-0357 and WVDC-0496 in an acute pneumonia model, six-week old female CD-1 mice were IP administered either an IgG2b isotype control mAb, *Pa* WCV serum, WVDC-0357, or WVDC-0496. After 12 h, mice were IN challenged with a 2x10^7^ CFU dose of *P. aeruginosa* PAO1. Mice were euthanized at 16 h post-challenge with an IP injection of Euthasol (390 mg/kg). Blood was collected *via* cardiac puncture and placed in serum separator tubes. The lung was aseptically removed and weighed. The right lobe of the lung was placed in a gentleMACS C tube and homogenized. The nasal wash was collected by injecting 1 mL of PBS through the nasal cavity. The homogenized lung and nasal wash were serially diluted, plated on PIA, and incubated overnight at 37 °C for CFU enumeration.

All animal care and use procedures in this study was in compliance with the National Institutes of Health Guide for the Care and Use of Laboratory animals. The West Virginia University Institutional Animal Care and Use Committee (WVU-ACUC protocol #1606003173) granted approval for all animal protocols.

### 2.10 Pharmacokinetics

To assess the pharmacokinetic profile of WVDC-0357 and WVDC-0496, nine-week old CD-1 mice were IP administered WVDC-0357 or WVDC-0496 at 45 mg/kg. There were 3 mice per group. Mice were submandibular bled at 1, 3, 7, 14, and 21 d post-administration of mAb. Sera was pooled for each time point and anti-IgG titers against *P. aeruginosa* PAO1 were determined by ELISA methods described above. The half-lives of WVDC-0357 and WVDC-0496 were determined using a one-phase exponential decay model.

### 2.11 Histopathology

Mouse lungs were prepared and sent for histopathological analysis. Following challenge and euthanasia of mice in the pneumonia model, the left lobe of the lung was placed in 10% neutral buffered formalin. Paraffin-embedded lung sections with 5 µm thickness were mounted on glass slides and stained with (i) hematoxylin and eosin to assess for acute inflammation and (ii) Gram stain to assess for presence of bacilli. Lungs were scored by a pathologist blinded to experimental groups.

### 2.12 Measurement of cytokine and chemokine levels

The concentration of CXCL-1, TNF-α, IL-1β, IL-6, IL-10, and IFN-γ in the serum and lung supernatant was quantified using a Luminex multiplex assay (R&D). Procedures were performed according to manufacturer’s instructions.

### 2.13 Antibody sequencing

The variable region of WVDC-0357 and WVDC-0496 were sequenced using methods by Meyer et al. (2019). Briefly, total RNA was extracted using Quick-RNA Microprep Kit (Zymo, #R1051). The cDNA was synthesized using the SMARTScribe Reverse Transcriptase kit (Clontech, #639537) with a custom template switch oligonucleotide and chain-specific reverse primers. Touchdown PCR was used to amplify the synthesized cDNA with a universal forward primer and nested chain-specific reverse primers. The amplified PCR reaction was run on a 1% agarose gel and DNA was extracted using the QIAquick Gel Extraction Kit (Qiagen, #28704). The extracted DNA was Sanger sequenced using the IgG reverse primers.

### 2.14 Statistical analysis

All statistical analyses were performed using GraphPad Prism version 9. Survival data is presented as a Kaplan-Meier curve and analyzed by the log-rank (Mantel-Cox) test. All other data is presented as the mean ± standard deviation (SD). For comparison between two groups, a two-tailed student’s t-test was performed. For comparison between three or more groups, a one-way ANOVA with Dunnett’s multiple comparison or Kruskal-Wallis with Dunn’s multiple comparison was performed depending on the normality of the data set. P values less than 0.05 were considered statistically significant.

## 3 Results

### 3.1 Convalescent mice previously infected with *P. aeruginosa* PAO1 cultured on PIAAMV are protected against lethal challenge

**Figure 1:**
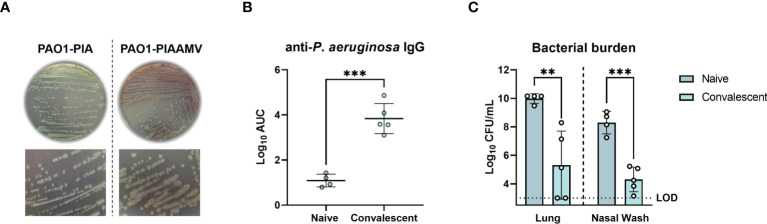
Protection of convalescent mice against lethal *P. aeruginosa* challenge after prior infection with *P. aeruginosa* PAO1 cultured on Pseudomonas isolation agar supplemented with ammonium metavanadate (PIAAMV). CD-1 mice were challenged with a sub-lethal intranasal dose (1.4x10^7^ CFU) of *P. aeruginosa* PAO1 cultured on PIAAMV and allowed to convalesce before being challenged with a lethal intranasal dose of *P. aeruginosa* PAO1 cultured on Pseudomonas isolation agar (PIA). Mice were euthanized at 16 h post-challenge. **(A)** Comparison of colony morphology between *P. aeruginosa* PAO1 cultured on PIA and *P. aeruginosa* PAO1 cultured on PIAAMV used for dose preparation. Growth of *P. aeruginosa* PAO1 on PIAAMV induced a mucoid colony morphology. **(B)** Serum anti-*P. aeruginosa* IgG titers determined by ELISA are represented by area under the curve (AUC) values. Differences in serum antibody titers were determined using a two-tailed student’s t-test. ***P<0.001 compared to naïve mice. **(C)** Bacterial burden in the lung and nasal wash was determined at 16 hours post-challenge. Differences in bacterial burden were determined using a two-tailed student’s t-test. **P<0.01 and ***P<0.001 compared to naïve mice. LOD, limit of detection.

We hypothesized that mice previously challenged with *P. aeruginosa* PAO1 cultured on PIAAMV would be protected from a lethal *P. aeruginosa* challenge. To test this hypothesis, *P. aeruginosa* PAO1 was cultured on PIAAMV (**Figure 1A**) and a sub-lethal dose (1.4x10^7^ CFU) was administered IN to six-week old female CD-1 mice, which were allowed to convalesce for 34 d. Naïve and convalescent mice were then challenged IN with a lethal dose of *P. aeruginosa* PAO1 cultured on PIA (1.4x10^8^ CFU), and were euthanized 16 h post-challenge to determine serum antibody titers and bacterial burden in the lungs and nasal cavity. Convalescent mice had significantly higher serum antibody titers, with a 2.62 log increase (P<0.0001) in anti-IgG antibody titers compared to naïve mice (**Figure 1B**). Furthermore, bacterial burden was significantly lower in convalescent challenged mice compared to naïve mice with a 4.68 log reduction in CFUs in the lungs (P<0.01) and a 3.98 log reduction in CFUs in the nasal wash (P<0.001) (**Figure 1C**). Overall, these findings demonstrate that mice previously exposed to *P. aeruginosa* PAO1 cultured on PIAAMV were protected against a lethal *P. aeruginosa* challenge and produced significantly greater anti-*P. aeruginosa* antibody responses compared to naïve mice. Based on these results, we hypothesized that immunization with *P. aeruginosa* cultured on PIAAMV would induce a robust production of anti-*P. aeruginosa* antibody-secreting cells that could be used for hybridoma generation.

### 3.2 Generation and characterization of monoclonal antibodies against *P. aeruginosa*

**Figure 2:**
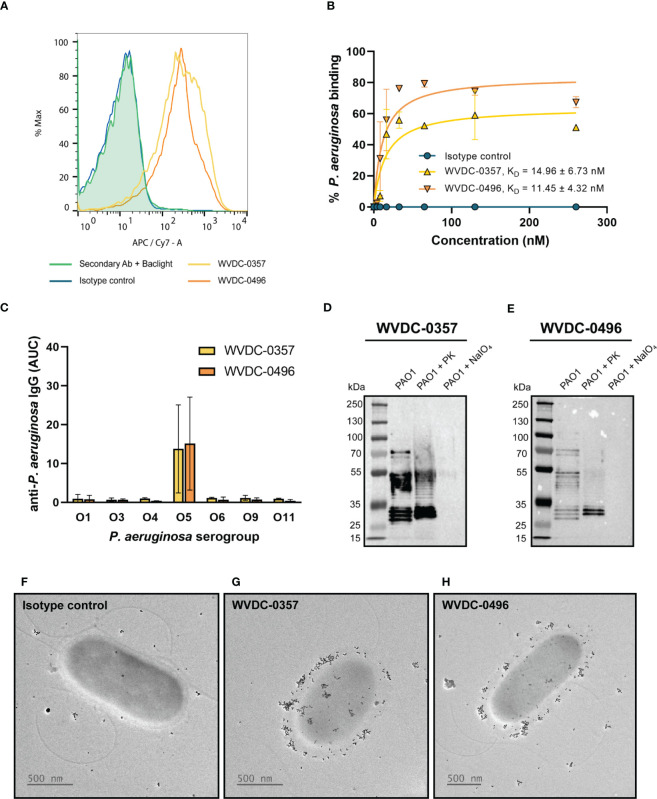
Characterization of anti-*P. aeruginosa* mAbs, WVDC-0357 and WVDC-0496. **(A)** Fluorescence-activated cell sorting (FACS) analysis of binding to *P. aeruginosa* was performed using WVDC-0357, WVDC-0496, and an isotype control mAb. **(B)** Binding kinetic analysis revealed dissociation constants (K_D_) of 14.96 ± 6.73 nM and 11.45 ± 4.32 nM for WVDC-0357 and WVDC-0496, respectively, using a non-linear regression model. **(C)** ELISA was used to determine anti-*P. aeruginosa* IgG titers against different serogroups, represented as area under the curve (AUC) values. **(D, E)** Immunoblot analysis of *P. aeruginosa* outer membrane fractions treated with proteinase K or sodium periodate, and probed with WVDC-0357 and WVDC-0496. **(F-H)** Electron microscopy imaging of *P. aeruginosa* treated with an isotype control mAb, WVDC-0357, or WVDC-0496, labeled with a gold-conjugated secondary antibody. PK, proteinase K; NaIO_4_, sodium periodate.

To generate mAbs against *P. aeruginosa*, CD-1 mice were immunized and boosted with live preparations of *P. aeruginosa* PAO1 grown on PIAAMV and following convalescence, hybridomas were formed by fusing spleen cells with immortalized myeloma cells. We screened a total of 508 hybridoma colonies and identified two IgG2b-kappa secreting hybridomas (WVDC-0357 and WVDC-0496) that had strong binding to intact *P. aeruginosa* PAO1 by ELISA (**Supplementary Figure 1**). WVDC-0357 and WVDC-0496 were subsequently purified for further characterization. Flow cytometry was performed to confirm binding of WVDC-0357 and WVDC-0496 to intact *P. aeruginosa* PAO1, which demonstrated an approximate 1-log shift in fluorescence above the isotype-matched control (**Figure 2A**). In addition, we showed that WVDC-0357 and WVDC-0496 had a dose-response profile of binding to *P. aeruginosa*. Binding kinetic studies were performed with flow cytometry, which demonstrated a dissociation constant (K_D_) in the nanomolar range. The obtained K_D_ values were 14.96 ± 6.73 nM and 11.45 ± 4.32 nM, for WVDC-0357 and WVDC-0496, respectively (**Figure 2B**, **Supplementary Table 2**). Sequencing of the antibody variable region revealed that WVDC-0357 and WVDC-0496 share similar variable regions with point mutations encoded in LFR3 and LFR4. For both antibodies, the light chain sequence was derived from the murine germline sequences IGKV1-122 and IGKJ2, and the heavy chain sequence was derived from the murine germline sequences IGHV1-52, IGHV1-61, IGHD1-1, and IGHJ4.

To further characterize the binding specificity of WVDC-0357 and WVDC-0496, an ELISA screen was conducted against a panel of *P. aeruginosa* strains (**Supplementary Table 1**), which included clinical strains obtained from Dr. Robert Ernst (Burns et al., 2001) and the Multidrug Resistant Organism Repository and Surveillance Network (MSRN) (Lebreton et al., 2021). The ELISA results showed that these mAbs bind specifically to *P. aeruginosa* strains from serogroup O5 (**Figure 2C**). To investigate the influence of stress conditions on binding, ELISAs were also conducted with each serogroup O5 strain cultivated on PIAAMV. There was a significant increase in the binding of WVDC-0357 and WVDC-0496 to serogroup O5 strains when cultivated on PIAAMV compared to PIA (**Supplementary Figure 2**). These findings suggest that WVDC-0357 and WVDC-0496 not only recognize serogroup O5 strains under standard growth conditions, but also demonstrate increased binding when these strains are exposed to stress induced by ammonium metavanadate.

Given the high specificity of WVDC-0357 and WVDC-0496 for serogroup O5 strains, we hypothesized that they targeted the polysaccharide component of *P. aeruginosa*. To test this hypothesis, a western blot was performed with *P. aeruginosa* PAO1 outer membrane fractions treated with proteinase K, to digest proteins, or sodium periodate, to degrade polysaccharides. The results of the western blot analysis indicated that WVDC-0357 and WVDC-0496 recognized polysaccharide as the antigen, as evidenced by the banding pattern observed (**Figures 2D, E**, **Supplementary Figure 3**). Furthermore, treatment of *P. aeruginosa* PAO1 with proteinase K did not alter binding, suggesting that the antibodies do not bind proteins; however, treatment of *P. aeruginosa* PAO1 with sodium periodate completely ablated binding, suggesting that these antibodies specifically bind polysaccharides (**Figures 2D, E**). In addition, we performed western blotting on a *P. aeruginosa* PAO1 transposon mutant deficient in the gene *wbpC*, which is involved in O-antigen biosynthesis and functions as a possible O-acetyltransferase (King et al., 2009). WVDC-0357 and WVDC-0496 did not bind to the transposon mutant for the gene *wbpC* (**Supplementary Figure 4**), further confirming that these antibodies target the O-antigen of LPS. Additional experiments were conducted against unrelated antigens, such as dextran, fetuin, and gelatin, however WVDC-0357 and WVDC-0496 did not show binding to these antigens (**Supplementary Figure 5**).

Lastly, we performed immunogold-labeling of *P. aeruginosa* to visualize surface binding by WVDC-0357 and WVDC-0496. Bacteria incubated with the isotype control mAb displayed no detectable surface binding of antibody (**Figure 2F**). In contrast, bacteria incubated with WVDC-0357 and WVDC-0496 demonstrated diffuse binding of antibody along the entire envelope surface (**Figures 2G, H**).

### 3.3 WVDC-0357 and WVDC-0496 induce bacterial aggregation

**Figure 3:**
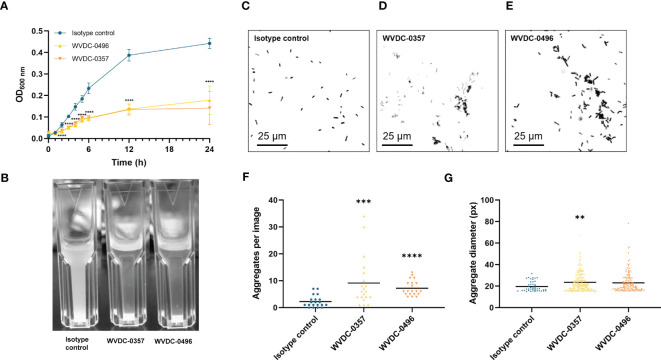
WVDC-0357 and WVDC-0496 promote bacterial agglutination. **(A)** In UV-transparent cuvettes, fresh cultures of *P. aeruginosa* PAO1 in lysogeny broth were incubated with an isotype control mAb, WVDC-0357, or WVDC-0496 at 100 µg/ml. Cuvettes were incubated statically at 37°C and the absorbance of growth cultures at 600 nm were measured over the course of 24 h. Differences in absorbance were analyzed using a one-way ANOVA with Dunnett’s multiple comparison test. *P<0.05, ***P<0.001, ****P<0.0001 compared to the isotype control. **(B)** After incubation for 24 h, cuvettes were photographed for visual inspection of turbidity. **(C-E)** Representative fluorescent microscopy images of *P. aeruginosa* PAO1 stained with BacLight Red and treated with an isotype control mAb, WVDC-0357, or WVDC-0496. **(F, G)** Random area images were analyzed with an aggregation cluster pipeline in CellProfiler for number of aggregates per image and aggregate diameter. Differences in aggregates were calculated using the Kruskal Wallis test with Dunn’s multiple comparison. **P<0.01, ***P<0.001, ****P<0.0001 compared to the isotype control.

Antibodies can play a critical role in the host defense against microbial pathogens through their canonical functions, such as aggregation and neutralization, complement binding, and opsonophagocytosis (Dimitrov and Lacroix-Desmazes, 2020). To investigate the functional properties of WVDC-0357 and WVDC-0496 in inducing aggregation of *P. aeruginosa*, bacterial growth curves were performed in UV-transparent cuvettes, whereby *P. aeruginosa* PAO1 was cultured in lysogeny broth with the inclusion of an isotype control mAb, WVDC-0357, or WVDC-0496. Cultures were incubated statically at 37°C and the absorbance at 600 nm was measured over a 24 h period. We observed a significant decrease in absorbance values in cultures containing WVDC-0357 and WVDC-0496 compared to the isotype control mAb, with significant differences (P<0.0001) at all time points starting at 2 h (**Figure 3A**). Furthermore, visualization of the cuvettes demonstrated clear differences in the turbidity of growth cultures at 24 h (**Figure 3B**). To determine if the decrease in turbidity was due to bacterial aggregation and precipitation, *P. aeruginosa* PAO1 was stained with BacLight red bacterial stain, treated with an isotype control mAb, WVDC-0357, or WVDC-0496, and visualized with fluorescence microscopy for bacterial aggregates. Visualization of fluorescent *P. aeruginosa* PAO1 treated with WVDC-0357 and WVDC-0496 demonstrated an increase in bacterial aggregates compared to the isotype control (**Figures 3C-E**). To quantify the number and size of bacterial aggregates, randomly imaged areas were analyzed for number of aggregates and mean aggregate diameter using CellProfiler. For WVDC-0357 and WVDC-0496, there were significant increases in the total number of aggregates per image compared to the isotype control (**Figure 3F**). Furthermore, the mean aggregate diameter was greater for WVDC-0357 and WVDC-0496 compared to the isotype control (**Figure 3G**). Additional experiments were performed to determine the role of WVDC-0357 and WVDC-0496 in mediating opsonophagocytosis and complement-mediated killing. However, data from these experiments suggest that these antibodies do not induce macrophage opsonophagocytosis or complement-mediated killing of *P. aeruginosa* under tested conditions (**Supplementary Figure 6**). Collectively, these results demonstrate that WVDC-0357 and WVDC-0496 induce bacterial aggregation but do not promote opsonophagocytic or complement-mediated killing of *P. aeruginosa*.

### 3.4 WVDC-0357 and WVDC-0496 mediate direct antibacterial activity against *P. aeruginosa*

**Figure 4:**
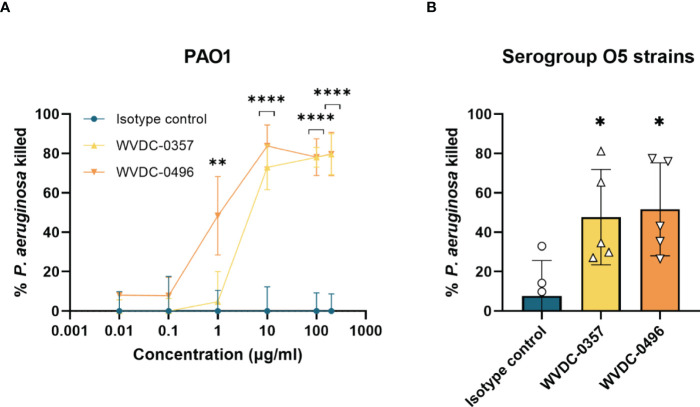
Antibacterial activity of WVDC-0357 and WVDC-0496. **(A)** Antibacterial activity against *P. aeruginosa* PAO1. Mid-log phase growth cultures of *P. aeruginosa* PAO1 were treated with varying concentrations of an isotype control mAb, WVDC-0357 or WVDC-0496 before plating onto Pseudomonas isolation agar (PIA) plates for CFU enumeration. The percent killing was determined by normalizing the number of CFUs from each sample to the mean CFUs of the no-antibody control. Differences in CFUs were calculated using a one-way ANOVA with Dunnett’s multiple comparison test. **P < 0.01, ****P<0.0001 compared to the isotype control. **(B)** Antibacterial activity against O5 serogroup strains. *P. aeruginosa* strains PAO1, MRSN 1601, MRSN 2101, MRSN 12368, and MRSN 14981 was treated with 100 µg/mL of an isotype control mAb, WVDC-0357, and WVDC-0496, and then plated on PIA for CFU enumeration. Each data point represents the average percent killing for a specific strain. The percent killing was determined by normalizing the number of CFUs from each sample to the mean CFUs of the no-antibody control. Differences in CFUs were calculated using a one-way ANOVA with Dunnett’s multiple comparison test. *P<0.05 compared to the isotype control.

Antibodies display a wide and diverse set of functions, yet recent studies have demonstrated the importance of the non-canonical function of antibodies in host immunity (Dimitrov and Lacroix-Desmazes, 2020). Besides the effector system pathways, antibodies are able to mediate antimicrobial activity through mechanisms such as direct pathogen inactivation (Dimitrov and Lacroix-Desmazes, 2020; Hotinger and May, 2020). To investigate the antibacterial activity of WVDC-0357 and WVDC-0496, bacterial killing assays were performed, whereby mid-log phase *P. aeruginosa* PAO1 was incubated with either an isotype control mAb, WVDC-0357, or WVDC-0496. After vortexing to ensure dissociation of any aggregates, the bacteria were plated for CFU enumeration. Results showed that WVDC-0357 and WVDC-0496 exhibited a dose-dependent killing effect on *P. aeruginosa* PAO1 with significant reductions in viable bacteria when compared to isotype control mAb-treated bacteria (**Figure 4A**). In addition, we evaluated the antibacterial activity of WVDC-0357 and WVDC-0496 against a diverse set of *P. aeruginosa* serogroup O5 strains, including strains sourced from the MRSN collection. This collection encompasses a wide range of *P. aeruginosa* strains, including extensively drug-resistant (XDR) strains (Lebreton et al., 2021). The efficacy of WVDC-0357 and WVDC-0496 varied among strains, yet collectively there was a significant increase in the percentage of *P. aeruginosa* killing compared to the isotype control (**Figure 4B**). Overall, these findings indicate that WVDC-0357 and WVDC-0496 effectively mediates direct bacterial killing of *P. aeruginosa*.

### 3.5 Pharmacokinetics of WVDC-0357 and WVDC-0496

**Figure 5:**
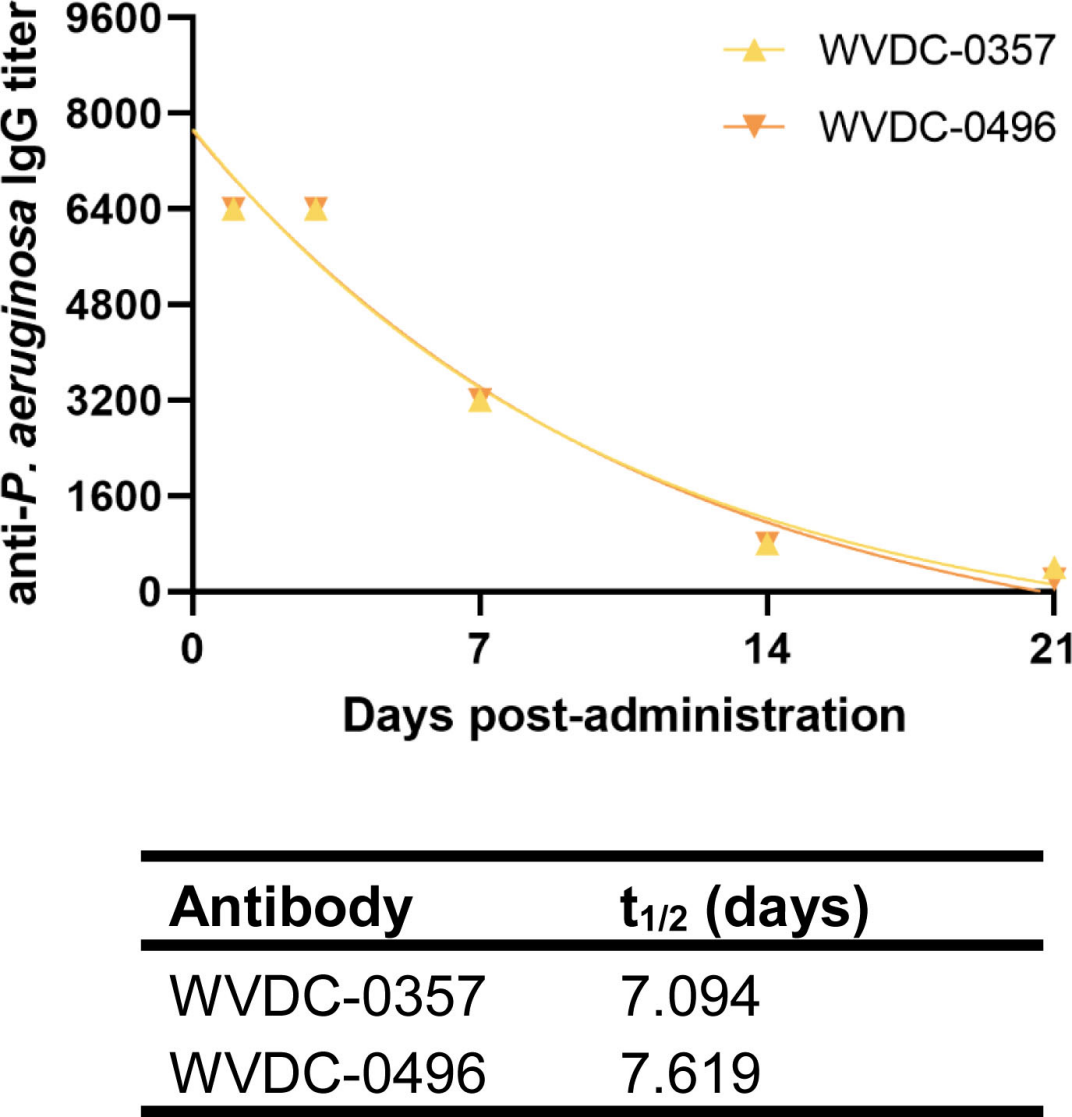
Pharmacokinetics of WVDC-0357 and WVDC-0496. CD-1 mice were intraperitoneally (IP) administered 45 mg/kg of WVDC-0357 or WVDC-0496 and submandibular bled at various time points over the course of 21 d to assess serum IgG titers against *P. aeruginosa* PAO1 using ELISA. The half-life (t_1/2_) of WVDC-0357 and WVDC-0496 was determined using a one-phase exponential decay model.

Determining the pharmacokinetic profiles of WVDC-0357 and WVDC-0496 is important to ensure adequate levels of these mAbs in circulation, allowing them to exert their target function. To assess the pharmacokinetics of WVDC-0357 and WVDC-0496, CD-1 mice were IP administered 45 mg/kg of antibody and were submandibular bled up to 21 d post-administration to collect serum. Antibody titers were determined using ELISA against intact *P. aeruginosa* PAO1 and plotted against time to determine their half-lives using a one-phase exponential decay model. Both WVDC-0357 and WVDC-0496 had similar pharmacokinetic profiles, with half-lives of 7.094 and 7.619 d, respectively (**Figure 5**). These half-life values are consistent with previous reports on the half-life of murine IgG2b antibodies (Vieira and Rajewsky, 1988). These findings indicate that WVDC-0357 and WVDC-0496 are stable in circulation for up to 72 h, providing insight for subsequent *in vivo* studies on the prophylactic administration of these mAbs.

### 3.6 WVDC-0357 and WVDC-0496 protect mice against lethal *P. aeruginosa* bloodstream challenge

**Figure 6:**
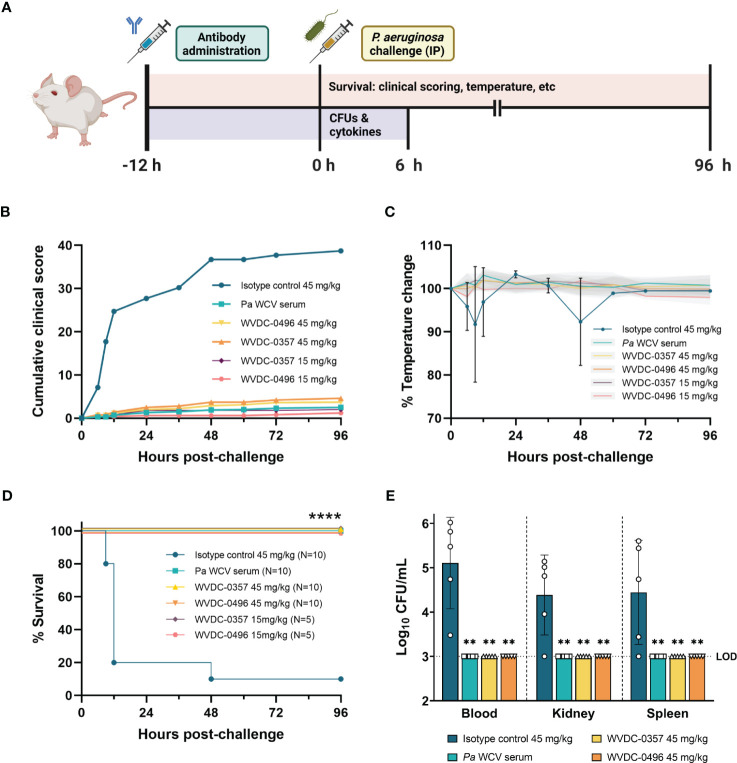
WVDC-0357 and WVDC-0496 protect mice following lethal bloodstream challenge with *P. aeruginosa*. **(A)** CD-1 mice were prophylactically administered either an isotype control mAb, *P. aeruginosa* whole cell vaccine (*Pa* WCV) serum, WVDC-0357, or WVDC-0496. After 12 h, mice were challenged with an intraperitoneal (IP) dose of *P. aeruginosa* PAO1 (5x10^5^ CFU) and survival was monitored for 96 h. A separate set of mice was euthanized at 6 h post-challenge to determine bacterial burden in the blood, kidney, and spleen. **(B)** In the survival model, clinical scores were calculated at the indicated time points. Shown is the cumulative total of average clinical scores for each group. **(C)** Temperature was a key indicator of morbidity in mice. Shown is the average percent temperature change of each group, with the standard deviation of the isotype control mice shown in black error bars and the standard deviation of all other groups shown as gray shaded regions. **(D)** The survival of mice is represented as Kaplan-Meier curves, with 5-10 mice in each group. Differences in survival were calculated using the log-rank test. ****P<0.0001 compared to isotype control. **(E)** At 6 h post-challenge, the blood, kidney, and spleen homogenate were serially diluted and plated to determine bacterial burden. Differences in CFUs were calculated using the Kruskal Wallis test with Dunn’s multiple comparison. **P<0.01 compared to the isotype control. Abbreviations: LOD, limit of detection.

Primary bloodstream infections are one of the most lethal complications of *P. aeruginosa* infections (Wisplinghoff et al., 2004). Given the functional profile of WVDC-0357 and WVDC-0496 against *P. aeruginosa*, we evaluated the efficacy of these mAbs in a lethal sepsis model. In this model, CD-1 mice were IP administered either an isotype control mAb, *P. aeruginosa* whole cell vaccinated (*Pa* WCV) serum as a positive control (Sen-Kilic et al., 2021; Blackwood et al., 2022), WVDC-0357, or WVDC-0496, and challenged 12 h later with a lethal IP dose of *P. aeruginosa* PAO1 (5x10^5^ CFU) (**Figure 6A**). Survival was monitored over the course of 96 h, and the severity of disease was assessed by scoring the mice daily based on clinical outcomes such as appearance, activity, eye closure, respiration quality, body temperature, and body weight. Each variable was given a score ranging from 0 to 4, and mice that had a total cumulative score of 12 or a score of 4 in any individual category were humanely euthanized. During the 96 h observation period, mice in the isotype control group quickly showed an increase in clinical scores, with a majority requiring euthanasia between 6 and 12 h post-challenge (**Figure 6B**, **Supplementary Figure 7**). As a secondary metric of overall health, temperature was a key indicator of morbidity. Mice that did not survive had on average a rectal temperature of 31.5°C, which was a 15% decrease from baseline. In contrast, mice administered *Pa* WCV serum, WVDC-0357, or WVDC-0496 maintained baseline rectal temperatures throughout the whole observation period (**Figure 6C**, **Supplementary Figure 7**). When looking at survival of mice, the majority of mice treated with the isotype control mAb required euthanasia within the first 24 h, and there was only a 10% survival rate at the conclusion of the observation period (**Figure 6D**). In contrast, mice treated with *Pa* WCV serum, 45 mg/kg of WVDC-0357, or 45 mg/kg of WVDC-0496 were fully protected and had a 100% survival rate (**Figure 6D**). This study was repeated with the addition of a 15 mg/kg dosing group for both WVDC-0357 and WVDC-0496, and these mice still demonstrated a 100% survival rate (**Figure 6D**).

**Figure 7:**
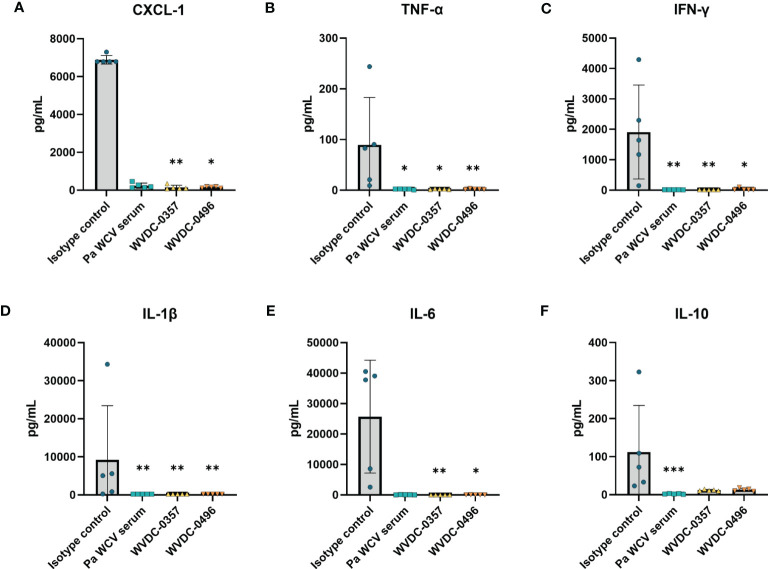
Level of inflammatory cytokines and chemokines in serum following lethal bloodstream challenge with *P. aeruginosa*. CD-1 mice were prophylactically administered an isotype control mAb, *P. aeruginosa* whole cell vaccine (*Pa* WCV) serum, WVDC-0357, or WVDC-0496, and challenged 12 h later with an intraperitoneal (IP) dose of *P. aeruginosa* PAO1 (5x10^5^ CFU). Mice were euthanized at 6 h post-challenge, and the serum concentration of **(A)** CXCL-1, **(B)** IFN-γ, **(C)** TNF-α, **(D)** IL-1β, **(E)** IL-6, and **(F)** IL-10 was measured with the Luminex assay. Differences in cytokines and chemokines were calculated using the Kruskal Wallis test with Dunn’s multiple comparison. *P<0.05, **P<0.01, ***P<0.001 compared to the isotype control.

To quantify the level of bacterial burden and inflammatory cytokines in the early stages of infection, an additional set of mice were euthanized at 6 h post-challenge. WVDC-0357 and WVDC-0496 significantly reduced bacterial burden to the limit of detection in the blood, kidney, and spleen, with a reduction in CFUs similar to the positive control *Pa* WCV serum group (**Figure 6E**). Compared to the isotype control mAb group, protected mice had a 2.11, 1.38, and 1.44 fold log reduction in CFUs in the blood, kidney, and spleen, respectively. The level of inflammatory cytokines and chemokines was drastically reduced in mice treated with WVDC-0357 and WVDC-0496 compared to isotype control mice (**Figures 7A-E**). The concentration of cytokines and chemokines was similar between the positive control *Pa* WCV serum group and WVDC-0357 and WVDC-0496, which was near the lower limit of detection. WVDC-0357 and WVDC-0496 significantly reduced the levels of CXCL-1, IFN-γ, TNF-α, IL-1β and IL-6 compared to the isotype control mAb (**Figures 7A-E**).

### 3.7 WVDC-0357 and WVDC-0496 protect mice against acute *P. aeruginosa* lung challenge

**Figure 8:**
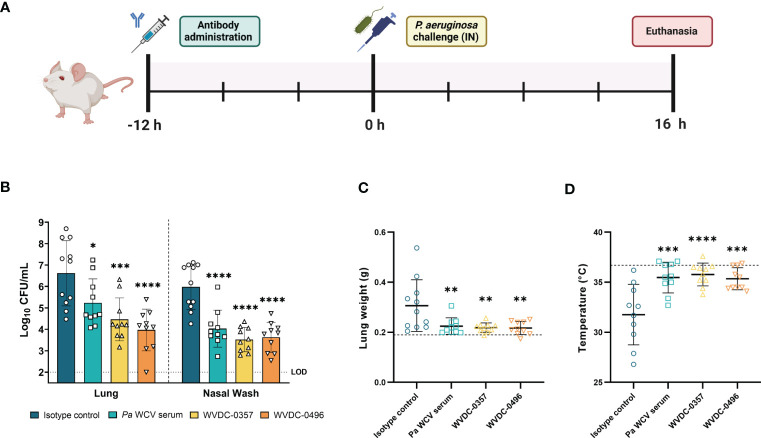
WVDC-0357 and WVDC-0496 enhance bacterial clearance in an acute pneumonia model. **(A)** CD-1 mice were prophylactically administered either an isotype control mAb, *P. aeruginosa* whole cell vaccine (*Pa* WCV) serum, WVDC-0357, or WVDC-0496, and after 12 h were challenged with an intranasal (IN) dose of *P. aeruginosa* PAO1 (2x10^7^ CFU). Mice were euthanized at 16 h post-challenge. **(B)** Bacterial burden in the lung and nasal wash of mice. Differences in CFUs were calculated using a one-way ANOVA with Dunnett’s multiple comparison test. *P<0.05, ***P<0.001, ****P<0.0001 compared to the isotype control. **(C)** Lung weights of mice were recorded at euthanasia. The dashed line represents the lung weight of naïve, non-challenged mice. Differences in lung weight were calculated using a one-way ANOVA with Dunnett’s multiple comparison test. **P<0.01 compared to the isotype control. **(D)** Rectal temperatures of mice at were recorded at euthanasia. The dashed line represents the rectal temperature of naïve, non-challenged mice. Differences in temperature were calculated using a one-way ANOVA with Dunnett’s multiple comparison test. ***P<0.001, ****P<0.0001 compared to the isotype control. Abbreviations: LOD, limit of detection.

We next evaluated the efficacy of WVDC-0357 and WVDC-0496 in an acute pneumonia model. CD-1 mice were IP administered either an isotype control mAb, *Pa* WCV serum, WVDC-0357, or WVDC-0496. After 12 h, mice were IN challenged with *P. aeruginosa* PAO1 (2x10^7^ CFU) and euthanized at 16 h post-challenge to assess for correlates of protection (**Figure 8A**). Our results showed that WVDC-0357 and WVDC-0496 significantly reduced bacterial burden in the respiratory tract of the mice. Compared to mice treated with an isotype control mAb, mice treated with WVDC-0357 had a 2.16 log reduction in lung CFUs (P<0.001) and 2.46 log reduction in nasal wash CFUs (P<0.0001), while mice treated with WVDC-0496 had a 2.71 log reduction in lung CFUs (P<0.0001) and a 2.35 log reduction in nasal wash CFUs (P<0.0001) (**Figure 8B**). This reduction in bacterial burden was similar to that observed in mice treated with *Pa* WCV serum. Wet lung weight was used as a marker of lung edema and we found a significant decrease in the lung weight of mice treated with *Pa* WCV serum, WVDC-0357, and WVDC-0496 when compared to the isotype control (**Figure 8C**). Additionally, the temperature of the mice was recorded as an overall metric of health. The average temperature in mice treated with *Pa* WCV serum, WVDC-0357, or WVDC-0496 was close to the physiological temperatures of non-challenged mice, with only a 2-3% decrease (**Figure 8D**). In contrast, mice treated with the isotype control mAb had an average decrease in rectal temperature of 13% when compared to the physiological temperature of non-challenged mice (**Figure 8D**).

### 3.8 WVDC-0357 and WVDC-0496 reduce lung inflammation post-challenge

**Figure 9:**
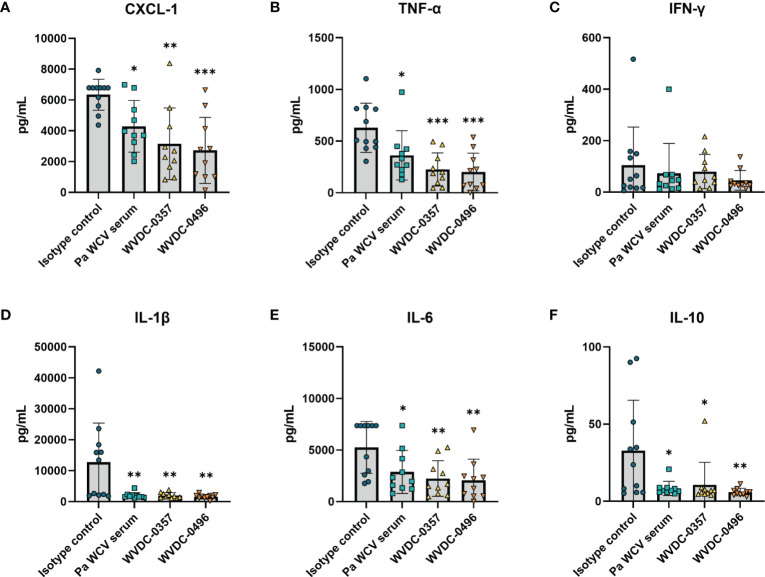
Level of inflammatory cytokines and chemokines in the lung following acute pneumonia challenge. CD-1 mice were prophylactically administered either an isotype control mAb, *Pa* WCV serum, WVDC-0357, or WVDC-0496, and challenged 12 h later with an intranasal dose of *P. aeruginosa* PAO1 (2x10^7^ CFU). Mice were euthanized 16 h post-challenge and the concentration of **(A)** CXCL-1, **(B)** IFN-γ, **(C)** TNF-α, **(D)** IL-1β, **(E)** IL-6, and **(F)** IL-10 in the lung supernatant was measured using the Luminex assay. Differences in cytokines and chemokines were calculated using a one-way ANOVA with Dunnett’s multiple comparison test. *P<0.05, **P<0.01, ***P<0.001, ****P<0.0001 compared to the isotype control.

**Figure 10:**
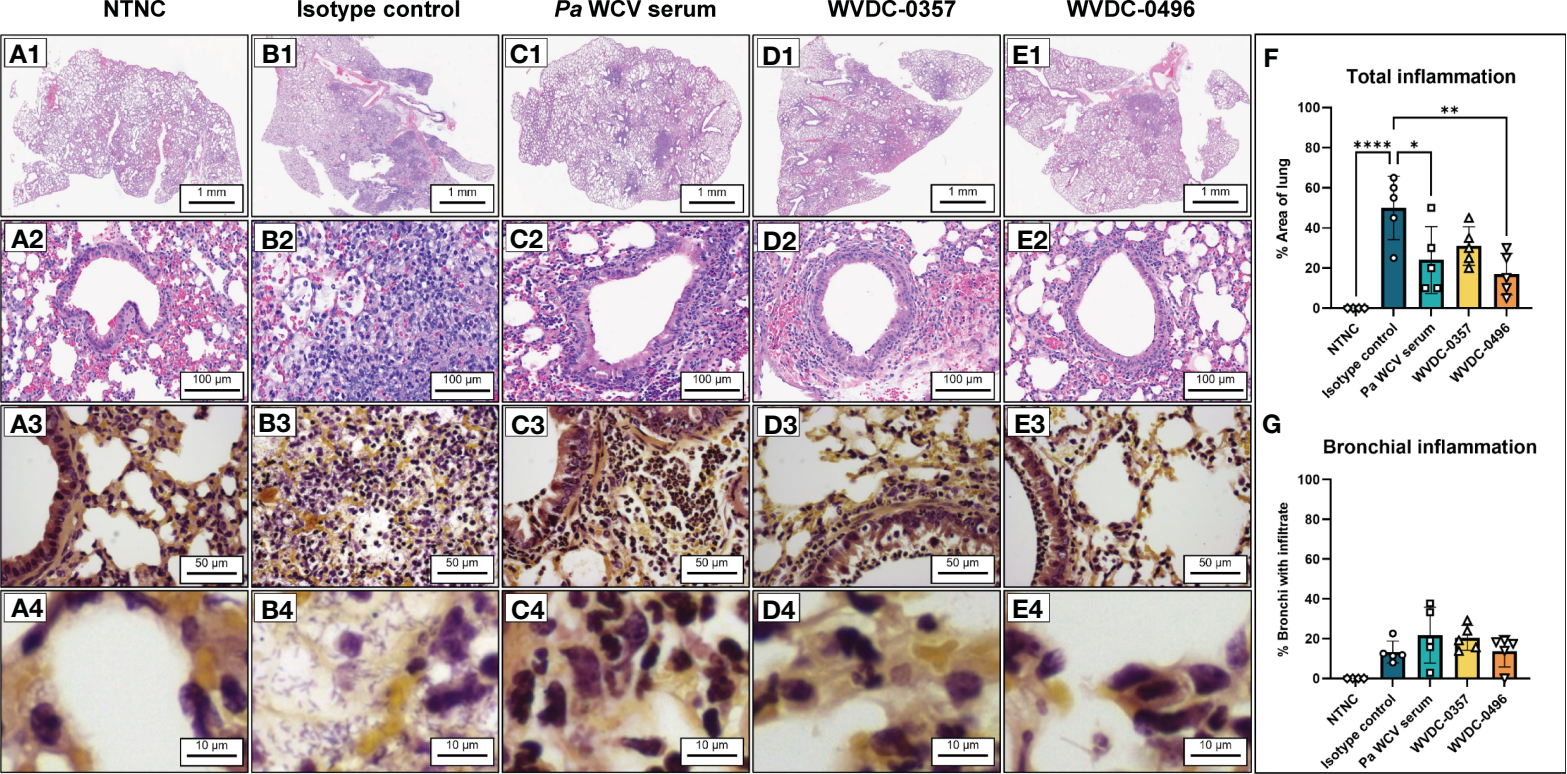
Representative hematoxylin and eosin (H&E) and Gram-stained sections obtained from mouse lungs following acute pneumonia challenge. **(A)** Lung from non-treated, non-challenged (NTNC) mouse showing **(A1)** unremarkable pulmonary parenchyma with no inflammation **(A2)** normal lung architecture with no alveolar or peribronchial acute inflammation, and **(A3, A4)** Gram stain revealing an absence of bacteria and inflammatory cells in the alveoli, bronchial lumen and peribronchial areas. **(B)** Lung from isotype control mAb-treated mouse displaying **(B1)** diffuse acute inflammation, **(B2)** acute inflammation of alveoli characterized by loose sheets of neutrophils, and **(B3, B4)** Gram stain revealing aggregates of bacilli in the alveoli and interstitium. **(C)** Lung from *P. aeruginosa* whole cell vaccine (*Pa* WCV) serum-treated mouse showing **(C1)** decreased net inflammation with inflammation largely restricted to peribronchial zones, **(C2)** acute inflammation surrounding bronchioles with relative preservation of bronchioles, and **(C3, C4)** Gram stain revealing an absence of bacilli in alveoli, interstitium and bronchi with surrounding peribronchial neutrophils. **(D)** Lung from WVDC-0357 treated mouse displaying **(D1)** patchy peribronchial inflammation, **(D2)** peribronchial acute inflammation similar to *Pa* WCV serum mice, and **(D3, D4)** Gram stain revealing an absence of bacilli in alveoli and bronchus and a layer of peribronchial neutrophils. **(E)** Lung from WVDC-0496 treated mouse demonstrating **(E1)** reduced acute inflammation restricted to rare peribronchial zones, **(E2)** peribronchial acute inflammation with rare, minor intraluminal infiltrate, and **(E3, E4)** Gram stain revealing an absence of bacilli and some neutrophils present along bronchial wall. **(F)** Mean lung area affected by inflammation across all groups. Differences in mean area of inflammation were calculated using a one-way ANOVA with Dunnett’s multiple comparison test. *P<0.05, **P<0.01, ****P<0.0001 compared to the isotype control. **(G)** Mean proportion of bronchial lumina affected by inflammation across all groups.

Acute lung infections caused by *P. aeruginosa* can trigger strong inflammatory cytokine and chemokine responses that are often associated with deleterious lung damage (Yanagihara et al., 2000; Mijares et al., 2011; Lin and Kazmierczak, 2017). We hypothesized that prophylactic treatment of mice with WVDC-0357 and WVDC-0496 would decrease key inflammatory markers and decrease total inflammation in the lung following intranasal challenge with *P. aeruginosa*. We found that mice treated with WVDC-0357 and WVDC-0496 had significantly lower levels of CXCL-1, TNF-α, IL-1β and IL-6, and IL-10 compared to mice treated with an isotype control mAb (**Figure 9**). Histopathological examination of the lungs demonstrated clear differences in the area and pattern of inflammation. In the absence of challenge, negative control mice displayed no signs of inflammation (**Figure 10A**). In contrast, mice in the isotype control group displayed a diffuse pattern of alveolar inflammation, characterized predominantly by neutrophils (**Figure 10B**). Gram staining revealed the presence of live bacilli with a diffuse distribution in all samples (**Figure 10B**). Among mice treated with *Pa* WCV serum, WVDC-0357, or WVDC-0496, there was a significant reduction in the mean area of inflammation (**Figures 10C–F**). Furthermore, inflammation was largely restricted to the peribronchial zones, migration through the pulmonary parenchyma was limited, and Gram staining revealed the absence of bacilli (**Figures 10C-E, G**). Taken together, these findings suggest that WVDC-0357 and WVDC-0496 are effective in mitigating key inflammatory markers and restricting the spread of inflammation in the lung against *P. aeruginosa* challenge.

## 4 Discussion

*P. aeruginosa* is one of the most commonly encountered nosocomial pathogens and is responsible for complications such as VAP and primary bloodstream infections (Spagnolo et al., 2021). Considering the prevalence of MDR strains of *P. aeruginosa* and mortality associated with these infections, there is an urgent need to develop alternative therapeutics against *P. aeruginosa*. In this study, we generated WVDC-0357 and WVDC-0496, which are highly specific to the O-antigen LPS of *P. aeruginosa.* Furthermore, we demonstrated that prophylactic administration of WVDC-0357 and WVDC-0496 is highly effective at protecting mice against lethal bacteremia and pneumonia.

We had previously demonstrated that when *P. aeruginosa* PAO1 is cultured on PIAAMV, a host of bacterial genes are differentially regulated when compared to PIA alone, including an upregulation in genes for LPS synthesis (Damron et al., 2013). Furthermore, convalescent mice previously infected with *P. aeruginosa* PAO1 cultured on PIAAMV were protected against a lethal *P. aeruginosa* challenge (**Figures 1B, C**). We hypothesized that the predominant antibody response triggered by *P. aeruginosa* PAO1 cultured on PIAAMV was primarily against outer polysaccharides and used convalescent mice who had been previously infected with *P. aeruginosa* PAO1 cultured on PIAAMV to raise therapeutic antibodies against *P. aeruginosa*. We utilized splenocytes derived from convalescent mice previously infected with *P. aeruginosa* PAO1 cultured on PIAAMV for hybridoma generation and identified two IgG2b mAbs, WVDC-0357 and WVDC-0496, that consistently screened positive against intact *P. aeruginosa* PAO1. Furthermore, sequencing of the variable regions of WVDC-0357 and WVDC-0496 revealed that these mAbs share the same germline genes, suggesting clonal selection of this particular epitope.

Through comprehensive ELISA and western blotting analysis, we demonstrated the specific binding of WVDC-0357 and WVDC-0496 to the O-antigen LPS of *P. aeruginosa* belonging to serogroup O5 (**Figures 2C, D**). LPS is a major component of the outer membrane and key virulence factor of *P. aeruginosa*, capable of eliciting inflammatory responses and inhibiting host-mediated bacterial defenses. The heterogeneity in the structure of the O-antigen component of LPS is the primary basis for serotyping schemes, which have evolved from traditional serological methods based on the International Antigenic Typing Scheme (IATS) (Liu et al., 1983; Liu and Wang, 1990) to next-generation sequencing-based approaches (Thrane et al., 2016). Based on the O-antigen gene cluster, *P. aeruginosa* can be classified into 12 serogroups, and several PCR-based methods have been developed for rapid serogroup identification (Li et al., 2018; Nasrin et al., 2022; Wang et al., 2022). Rapid identification of *P. aeruginosa* by its serogroup not only allows for targeted treatment approaches, but also mitigates the risk of antibiotic resistance associated with broad-spectrum antibiotics. Our findings highlight the potential therapeutic effect of WVDC-0357 and WVDC-0496 towards serogroup O5 strains, even demonstrating efficacy against antibiotic-resistant *P. aeruginosa* strains. Interestingly, WVDC-0357 and WVDC-0496 exhibited enhanced binding against serogroup O5 strains cultivated in stressed growth conditions induced by ammonium metavanadate. This highlights the potential of these antibodies to function in various environmental contexts, ranging from acute infection settings to chronic infection settings.

Based on the known prevalence of serogroup O5 strains, WVDC-0357 and WVDC-0496 could be utilized for the treatment or prevention of 5-10% of *P. aeruginosa* infections (Thrane et al., 2016; Freschi et al., 2019). The narrow binding spectrum of these antibodies is beneficial as it suggests that they could be administered with less off-target effects on the natural microbial populations. However, considering the need for a comprehensive therapeutic approach, we propose that creating combinations of antibodies that target most of the clinically relevant LPS serogroups of *P. aeruginosa* is a viable solution for expanding their application.

In this study, we demonstrated the efficacy of WVDC-0357 and WVDC-0496 in mitigating *P. aeruginosa* across multiple models of infection. When untreated mice are challenged with a lethal dose of *P. aeruginosa*, they typically become morbid and require humane euthanasia within 24 to 48 h, with pathogenicity driven in large part by endotoxic shock (Secher et al., 2011; DiGiandomenico et al., 2012). In our mouse models of infection, we demonstrated that prophylactic administration of WVDC-0357 and WVDC-0496 was able to improve survival of mice and significantly reduce bacterial burden (**Figures 6** and **8**). Limiting the spread of infection is invariably important in preventing the onset of lethal endotoxic shock. Dysregulation of pro-inflammatory cytokines (TNF-α, IL-1β and IL-6) and anti-inflammatory cytokines (IL-10) are often associated with life-threatening infection or presence of endotoxin. The chemokine CXCL-1 is associated with neutrophil recruitment and is important in combating early infection, although prolonged neutrophil accumulation is associated with deleterious inflammation (Sun et al., 2009). In our sepsis and pneumonia models, the cytokine profile suggested that WVDC-0357 and WVDC-0496 were able to attenuate severe infection and endotoxic shock from taking place (**Figures 7**, **9**). While the findings from our study provide valuable insights into the prophylactic use of WVDC-0357 and WVDC-0496, future studies will involve evaluating the efficacy of these mAbs in a therapeutic model to fully assess their potential as a treatment option for *P. aeruginosa* infections.

Our study aligns with previous research efforts that have developed anti-*P. aeruginosa* mAbs targeting the O-antigen of LPS. These studies demonstrated efficacy in terms of bacterial clearance and protection against infection in mouse models, however these mAbs were specifically directed against IATS serotypes O6 or O11 (Akiyama et al., 2000; Hemachandra et al., 2001; Horn et al., 2010; Xie et al., 2010; Que et al., 2014). In order to address the diverse strains of *P. aeruginosa* encountered in clinical settings, it becomes important to expand the armamentarium of antibodies to include a wider array of serotypes, such as O5. The development of WVDC-0357 and WVDC-0496 represents a significant contribution to the overall effort of providing a more comprehensive and effective antibody-based therapy against *P. aeruginosa* infections. In addition to the specificity for the O-antigen of LPS, our study revealed a unique aspect of these antibodies. Despite Fc effector functions being typically considered essential for antibacterial mAbs, WVDC-0357 and WVDC-0496 demonstrated direct bactericidal activity. While it has been reported that directly bactericidal antibodies usually target essential outer membrane proteins (Šadžiene et al., 1993; Šadžiene et al., 1994; Goel and Kapil, 2001; Storek et al., 2018), the identification of bactericidal antibodies that specifically target polysaccharides is less frequently reported. However, in a study by Yano et al., they identified that their directly bactericidal pneumococcal capsular polysaccharide mAbs against *Streptococcus pneumoniae* altered the quorum sensing response, which led to a modified bacterial agglutination and ultimately fratricide (Yano et al., 2011). We speculate that the binding of WVDC-0357 and WVDC-0496 to *P. aeruginosa* may similarly alter the quorum sensing response, leading to bacterial agglutination and fratricide. Additionally, the structure of LPS is influenced by its local ionic environment, with divalent cations stabilizing LPS in the outer membrane while monovalent cations destabilize the lipid bilayer and lead to pore formation (Adams et al., 2014). Thus, disrupting the interaction of divalent cations with LPS could disrupt the conformational and dynamic properties of LPS, leading to membrane destabilization. However, further investigation is needed to understand the potential interactions and mechanisms of our antibodies.

One limitation of our study is that WVDC-0357 and WVDC-0496 are murine antibodies, which may have implications in efficacy and safety when translated to human use. Potential issues of murine antibodies include shorter half-lives and the possibility of triggering human anti-mouse antibody (HAMA) responses (Samaranayake et al., 2009). To address these concerns, the variable regions of these antibodies would need to be humanized and expressed recombinantly, which would minimize the risk of immunogenicity and enhance the compatibility of these antibodies with the human immune system. In addition to humanization, it is important to adhere to regulatory guidelines to assess the safety profile of these antibodies before advancing to clinical trials. According to the FDA’s guidance on antibody production, pre-clinical testing would be required to evaluate potential cross-reactivity to different types of human tissue and to assess toxicity in animal models (Miele and Stein, 1997).

In summary, our study describes the development and characterization of novel mAbs directed against *P. aeruginosa* from serogroup O5. Our findings demonstrate that WVDC-0357 and WVDC-0496 are highly protective in both sepsis and pneumonia models of *P. aeruginosa* infection by promoting bacterial clearance and preventing endotoxic shock. Future work will focus on the translation of these mAbs towards clinical applications through humanization and additional translational studies. It is important to note that WVDC-0357 and WVDC-0496 only bind a subset of *P. aeruginosa* strains, and collaboration will be necessary to combine existing anti-LPS mAbs into a single therapy targeting the most prevalent serogroups of *P. aeruginosa*. Overall, these results have promising implications for the development of effective treatments against *P. aeruginosa* infections.

## Data availability statement

The original contributions presented in the study are included in the article/**Supplementary Materials**. Further inquiries can be directed to the corresponding authors.

## Ethics statement

The animal study was reviewed and approved by West Virginia University Institutional Animal Care and Use Committee (WVU-ACUC).

## Author contributions

JK, FD, and MB conceptualized and designed all experiments. WVDC-0357 and WVDC-0496 were generated and purified by JK, MM-B, and JC. Flow cytometry binding assays were conducted by JK, ES-K, and MN. Binding and functional assays were performed by JK and SM, while pharmacokinetic assays were carried out by JK and SD. Mice immunizations, administration of challenge doses, and health checks were performed by JK, MM-B, and SM. Mice necroscopy and tissue processing were conducted by JK, MM-B, SM, AH, GP, SD, and EY. Histopathological analysis was completed by HM and MA. JK, FD, and MB analyzed data and composed the manuscript. All authors reviewed and edited the manuscript.
