# Supplementary material for: Monoclonal antibodies against lipopolysaccharide protect against Pseudomonas aeruginosa challenge in mice
Source: Front Cell Infect Microbiol. 2023 Jun 22;13:1191806. doi: 10.3389/fcimb.2023.1191806 (PMC10326049; doi:10.3389/fcimb.2023.1191806)
Supplement: Supplementary file 1 [file DataSheet_1.docx]

**Supplementary Table 1.** **Bacterial strains used in this study.** Serogroups of clinical isolates were predicted using the *Pseudomonas aeruginosa* serotyper (PAst).

| **Bacterial Strain** | **Serogroup** | **Source** |
| --- | --- | --- |
| *P. aeruginosa* PAO1 Vasil | O5 | Barton et al., 1996 |
| *P. aeruginosa* CEC 31 | O9 | Burns et al., 2001 |
| *P. aeruginosa* CEC 32 | O5 | Burns et al., 2001 |
| *P. aeruginosa* CEC 34 | O1 | Burns et al., 2001 |
| *P. aeruginosa* CEC 44 | O1 | Burns et al., 2001 |
| *P. aeruginosa* CEC 45 | O6 | Burns et al., 2001 |
| *P. aeruginosa* CEC 55 | O11 | Burns et al., 2001 |
| *P. aeruginosa* CEC 60 | O6 | Burns et al., 2001 |
| *P. aeruginosa* CEC 75 | O4 | Burns et al., 2001 |
| *P. aeruginosa* CEC 79 | O5 | Burns et al., 2001 |
| *P. aeruginosa* CEC 86 | O3 | Burns et al., 2001 |
| *P. aeruginosa* MRSN 1601 | O5 | Lebreton et al., 2021 |
| *P. aeruginosa* MRSN 2101 | O5 | Lebreton et al., 2021 |
| *P. aeruginosa* MRSN 12368 | O5 | Lebreton et al., 2021 |
| *P. aeruginosa* MRSN 14981 | O5 | Lebreton et al., 2021 |
| *P. aeruginosa* PAO1 Washington | O5 | Held et al., 2012 |
| *P. aeruginosa* PAO1 Tn::*wbpC* |  | Held et al., 2012 |

**Supplementary Table 2.** **Statistical analysis of Figure 2B.** Mean and standard deviation of antibody binding to *P. aeruginosa* PAO1 using flow cytometry. The data correspond to those plotted in Figure 2B.

|  | Isotype control | | WVDC-0357 | | WVDC-0496 | |
| --- | --- | --- | --- | --- | --- | --- |
| Concentration (nM) | **Mean** | **SD** | **Mean** | **SD** | **Mean** | **SD** |
| 0 | 0 | 0 | 0.05 | 0.05 | 0 | 0 |
| 4 | 0.01 | 0.01 | 0.755 | 0.475 | 2.355 | 1.765 |
| 8 | 0.005 | 0.005 | 7.12 | 2.47 | 31.15 | 16.75 |
| 16 | 0.005 | 0.005 | 46.85 | 11.25 | 55.95 | 13.95 |
| 32 | 0.04 | 0.02 | 55.85 | 3.75 | 76.2 | 0.6 |
| 65 | 0.005 | 0.005 | 52.25 | 0.15 | 79.4 | 1.6 |
| 130 | 0.05 | 0.05 | 59 | 11.1 | 74.45 | 1.85 |
| 260 | 0.07 | 0.04 | 51.1 | 0.3 | 67.35 | 2.55 |


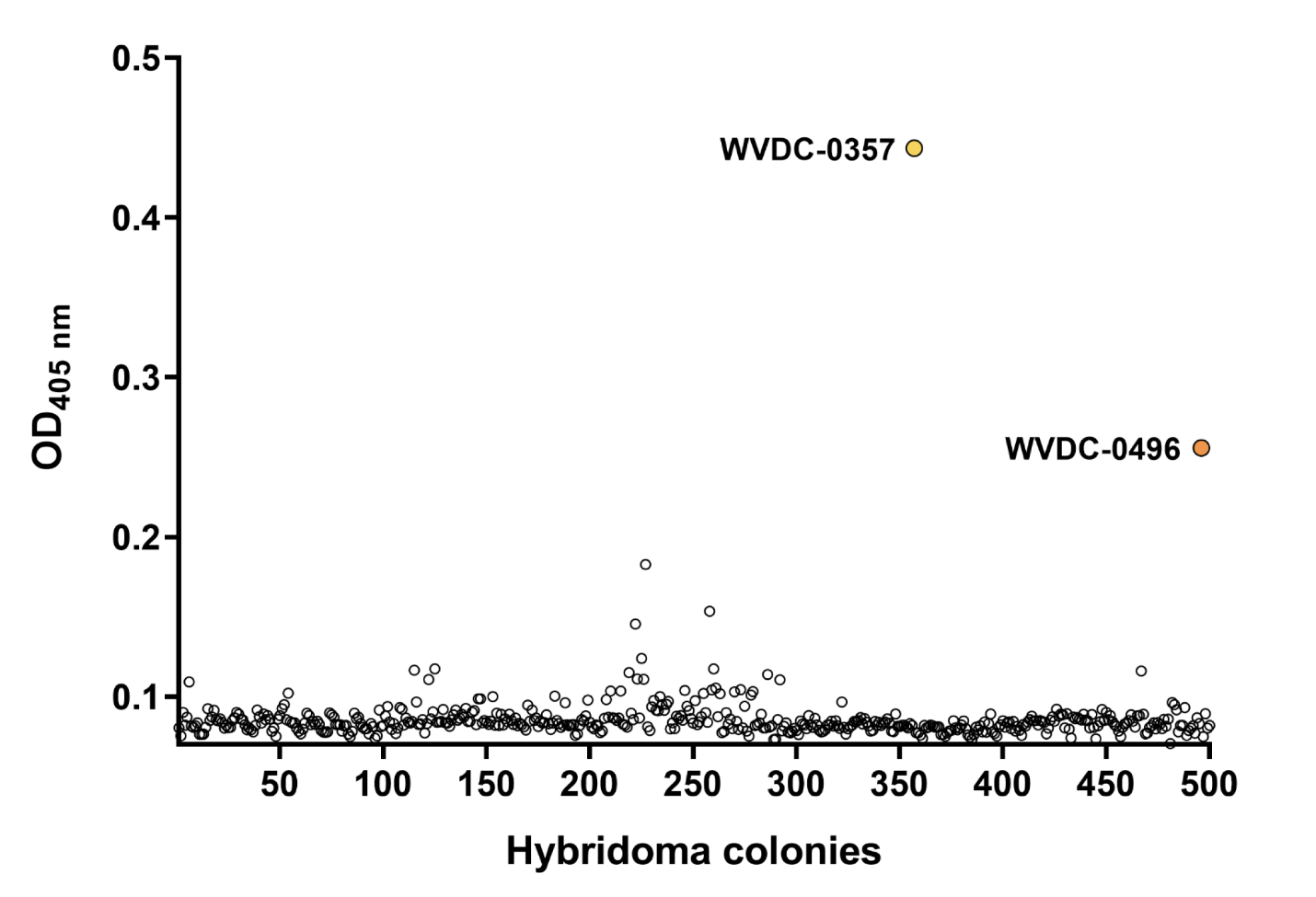


**Supplementary Figure 1.** **ELISA screening of hybridoma colonies against P. aeruginosa PAO1.** Hybridoma colonies WVDC-0357 and WVDC-0496 were selected based on their positive binding to whole bacteria.


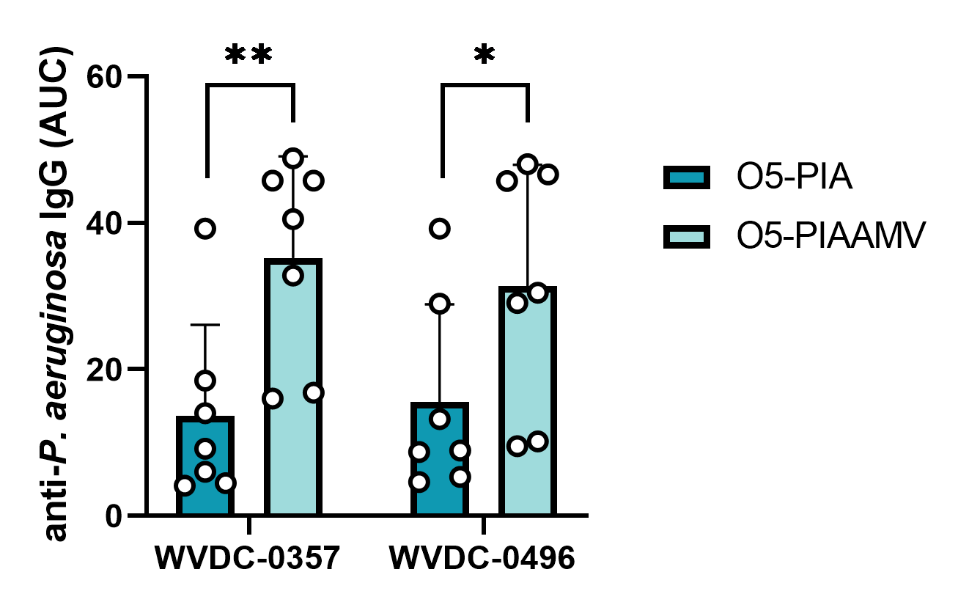


**Supplementary Figure 2.** **ELISA analysis of WVDC-0357 and WVDC-0496 binding to serogroup O5 strains grown on PIA and PIAAMV.** *P. aeruginosa* strains PAO1, MRSN 1601, MRSN 2101, MRSN 12368, MRSN 14981, CEC 32, and CEC 79 were cultured on both Pseudomonas isolation agar (PIA) and PIA supplemented with ammonium metavanadate (PIAAMV). An ELISA was performed to evaluate the binding of WVDC-0357 and WVDC-0496 to these strains. The results are presented as the area under the curve (AUC). Each data point represents the average AUC for a specific strain. Differences in AUC were calculated using a two-tailed student’s t-test. Significance levels were denoted as *P<0.05 and **P<0.01.


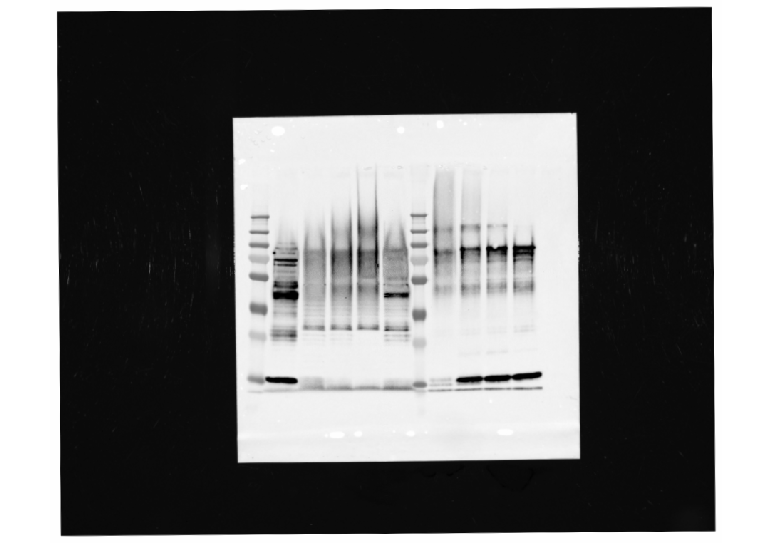


**250**

**130**

**100**

**70**

**55**

**35**

**25**

**15**

**kDa**

**PAO1**

**PAO1 + PK (16 h)**

**PAO1 + PK (2 h)**

**PAO1 + PK (30 min)**

**PAO1 + PK (1 min)**

**PAO1 + NaIO_4_ (16 h)**

**PAO1 + NaIO_4_ (2 h)**

**PAO1 + NaIO_4_ (30 min)**

**PAO1 + NaIO_4_ (1 min)**

**Supplementary Figure 3. Western blot analysis of *P. aeruginosa* PAO1 treated with proteinase K (PK) or sodium periodate (NaIO_4_).** *P. aeruingosa* PAO1 was treated with 0.2 mg/mL PK or 20 mM NaIO_4_ at 37 °C for 1 min, 30 min, 2 h, and 16 h, and western blot analysis was performed using *P. aeruginosa* whole cell vaccine serum to assess degradation of the targeted antigens.


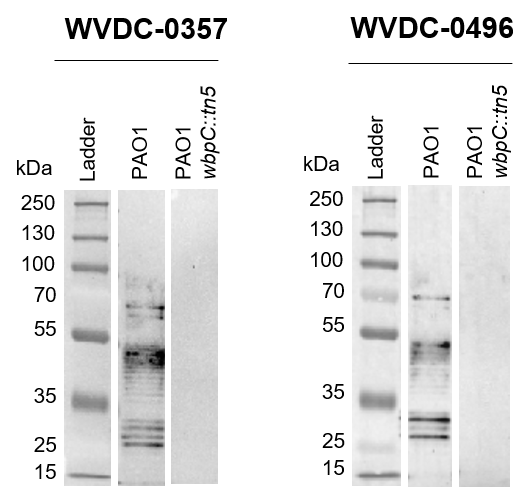


**Supplementary Figure 4.** **Western blot analysis of a PAO1 transposon mutant of LPS using WVDC-0357 and WVDC-0496.** Outer membrane fractions were prepared from *P. aeruginosa* PAO1 Washington and *P. aeruginosa* PAO1 Tn::*wbpC*, and probed with WVDC-0357 and WVDC-0496.


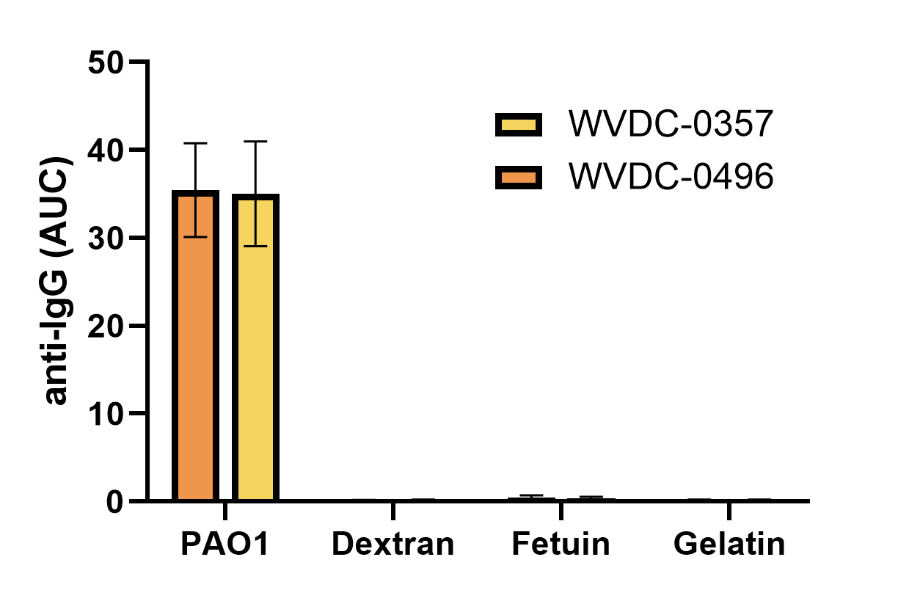


**Supplementary Figure 5.** **Binding specificity of WVDC-0357 and WVDC-0496 against dextran, fetuin, and gelatin.** An ELISA was conducted to assess the binding of WVDC-0357 and WVDC-0496 to the antigens dextran, fetuin, and gelatin. The results are presented as the area under the curve (AUC). The AUC against PAO1 is provided as a reference for comparison.


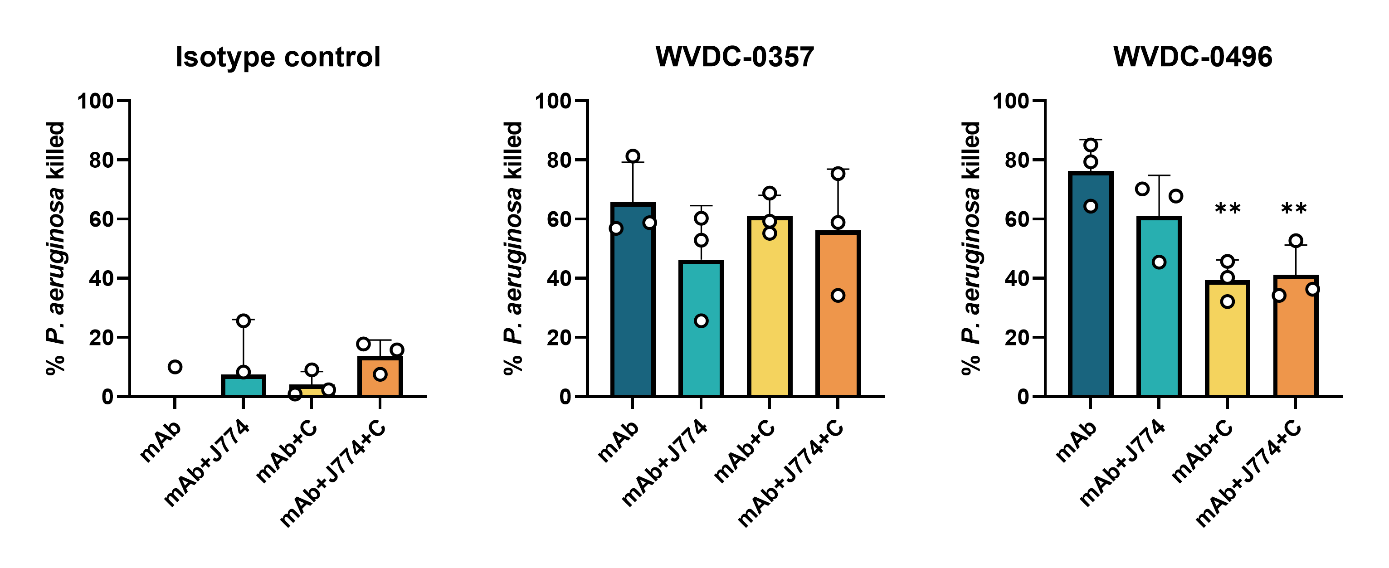


**Supplementary Figure 6.** **Antibacterial activity of WVDC-0357 and WVDC-0496 in the presence of macrophages and complement.** *P. aeruginosa* PAO1 was cultured to mid-log phase growth and incubated with either an isotype control mAb, WVDC-0357 or WVDC-0496 at 100 µg/ml. After incubation for 1 h at room temperature, opsonization buffer B, J774A.1 macrophages, baby rabbit complement, or J774A.1 macrophages with baby rabbit complement were added to the bacterial-antibody complexes and allowed to incubate for 1 h at 37 °C. After incubation, samples were serially diluted and plated on Pseudomonas isolation agar (PIA) for CFU enumeration. Percent killing was calculated by normalizing the number of CFUs from each sample to the mean CFUs of a no mAb control. Differences in percent killing was calculated using one-way ANOVA with Dunnett’s multiple comparison test. **P<0.01 compared to mAb-only group. Abbreviations: J774, J774A.1 macrophage; C, baby rabbit complement.

**
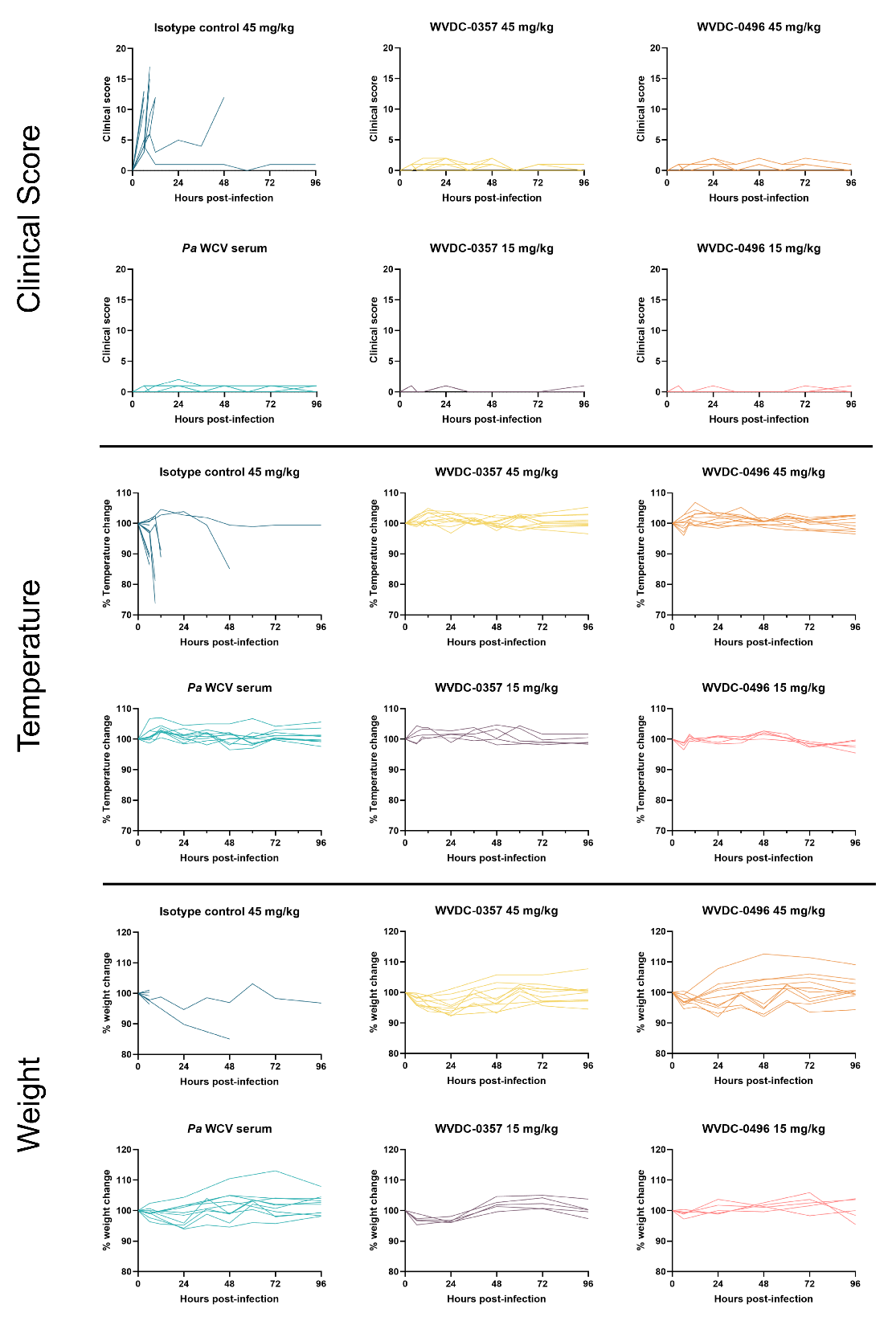
**

**Supplementary Figure 7.** **Clinical scoring, temperature, and weight of mice following lethal bloodstream challenge with *P. aeruginosa*.** Mice were administered either an isotype control mAb, *Pa* WCV serum, WVDC-0357, or WVDC-0496. After 12 h, mice were intraperitoneally (IP) challenged with a lethal dose of *P. aeruginosa* PAO1 and monitored for survival. Daily assessments of mice were performed, including evaluation of appearance, activity, eye closure, respiration quality, body temperature, and body weight loss. For each category, mice were scored from 0-4, where 0 represented no symptoms and 4 represented the most severe phenotype. Each line represents the clinical scoring, temperature, and weight of an individual mouse.
